# Reduced-Cost Four-Component Relativistic Double Ionization Potential Equation-of-Motion Coupled-Cluster Approaches with 4‑Hole–2-Particle Excitations and Three-Body Clusters

**DOI:** 10.1021/acs.jctc.5c01791

**Published:** 2026-03-27

**Authors:** Tamoghna Mukhopadhyay, Madhubani Mukherjee, Karthik Gururangan, Piotr Piecuch, Achintya Kumar Dutta

**Affiliations:** † Department of Chemistry, 29491 Indian Institute of Technology Bombay, Powai, Mumbai 400076, India; ‡ Department of Chemistry, 3078 Michigan State University, East Lansing, Michigan 48824, United States; § Department of Physics and Astronomy, 3078 Michigan State University, East Lansing, Michigan 48824, United States; ∥ Department of Inorganic Chemistry, Faculty of Natural Sciences, Comenius University, Ilkovičova 6, 84215 Mlynská dolina, Bratislava, Slovakia

## Abstract

The double ionization potential (DIP) equation-of-motion (EOM) coupled-cluster (CC) method with 4-hole–2-particle (4*h*-2*p*) excitations on top of the CC with singles, doubles, and triples calculation, abbreviated as DIP-EOMCCSDT­(4*h*-2*p*), along with its perturbative DIP-EOMCCSD­(T)­(a)­(4*h*-2*p*) approximation, are extended to a relativistic four-component (4c) framework. In addition, we introduce and test a new computationally practical DIP-EOMCC approach, which we call DIP-EOMCCSD­(T)­(*ã*)­(4*h*-2*p*), that approximates the treatment of 4*h*-2*p* correlations within the DIP-EOMCCSD­(T)­(a)­(4*h*-2*p*) method and reduces the $N^{8}$ scaling characterizing DIP-EOMCCSDT­(4*h*-2*p*) and DIP-EOMCCSD­(T)­(a)­(4*h*-2*p*) to $N^{7}$ with the system size N . Further improvements in computational efficiency are obtained using the frozen natural spinor (FNS) approximation to reduce the numbers of unoccupied spinors entering the correlated steps of the DIP-EOMCC calculations according to a well-defined occupation-number-based threshold. The resulting 4c-FNS-DIP-EOMCC approaches are used to compute DIPs for the series of inert gas atoms from argon to radon as well as the vertical DIPs in Cl_2_, Br_2_, HBr, and HI, which have been experimentally examined in the past. We demonstrate that, when using complete basis set extrapolations and FNS truncation threshold of 10^–4.5^, the 4c-FNS-DIP-EOMCCSD­(T)­(*ã*)­(4*h*-2*p*) calculations are capable of predicting DIPs in agreement with experimental data, improving upon their nonrelativistic and spin-free scalar-relativistic counterparts, particularly when examining DIPs characterized by stronger spin–orbit coupling effects.

## Graphical abstract

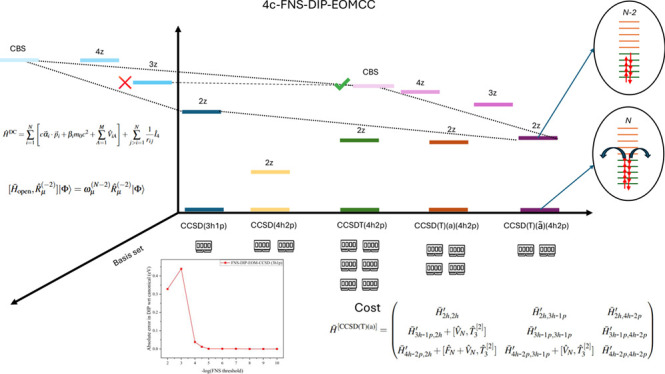

## 1 Introduction

The accurate treatment of relativistic effects in chemical systems has become an increasingly important facet of modern computational chemistry. One application of relativistic quantum chemical methods is the prediction of double ionization potentials (DIPs), which are critical to understanding photoelectron and Auger electron spectroscopies. Indeed, spin–orbit coupling and other relativistic contributions can significantly impact core and valence double ionization spectra in systems containing heavier atoms.^1,2^ The accurate prediction of DIPs in such systems remains a challenging problem for many electronic structure methods due to the need to treat relativistic interactions while capturing and balancing the many-electron correlation effects characterizing the *N*- and (*N* – 2)-electron species.^3–9^ While scalar-relativistic methods, such as the zero-order regular approximation (ZORA),^10–12^ Douglas–Kroll–Hess (DKH) transformations,^13–15^ and spin-free exact two-component (SFX2C) frameworks,^16,17^ are widely used, more complete four-component (4c) approaches are preferable and can serve as high-quality references, especially when dealing with stronger relativistic effects.

Unfortunately, the application of 4c electron correlation methods for describing double ionization suffers from increased computational costs due to the use of spinor bases, complex-valued Hamiltonians and wave functions, and the lack of spin *S* ^2^ and *S*_*z*_ symmetries. The use of uncontracted (e.g., Dyall-type^18,19^ ) basis sets further exacerbates this issue. In this work, we address this challenge by developing *ab initio* 4c approaches capable of obtaining accurate DIPs in a computationally practical fashion.

Among the various techniques for computing DIPs in many-electron systems, the equation-of-motion (EOM) coupled-cluster (CC) method offers an excellent balance between accuracy and computational cost, allowing one to recover the exact, full configuration interaction (CI) results using a systematically improvable hierarchy of approximations that can be performed using polynomial computational steps. As recently demonstrated in ref 20, the inclusion of one-, two-, and three-body clusters along with 2-hole (2*h*), 3-hole–1-particle (3*h*-1*p*), and 4-hole–2-particle (4*h*-2*p*) excitations in the DIP-EOMCC calculations, corresponding to the approach abbreviated as DIP-EOMCCSDT­(4*h*-2*p*), provides a highly accurate description of DIPs due to, in large part, achieving a well-balanced treatment of the correlation effects characterizing the *N*- and (*N* – 2)-electron states. In order to reduce the costs of the high-level DIP-EOMCCSDT­(4*h*-2*p*) calculations, which scale as $N^{8}$ with the system size N , ref 20 also introduced the perturbative DIP-EOMCCSD­(T)­(a)­(4*h*-2*p*) approximation, which avoids the expensive CC calculation with singles, doubles, and triples (CCSDT),^21–24^ used to describe the *N*-electron ground-state, by accounting for the effects of three-body clusters using perturbative arguments inspired by the CCSD­(T)­(a)-based approach of ref 25. It was demonstrated that the DIP-EOMCCSD­(T)­(a)­(4*h*-2*p*) method accurately reproduces the DIPs obtained using its DIP-EOMCCSDT­(4*h*-2*p*) parent for several diatomic molecules near their equilibrium geometries.

Given the computational challenges associated with the 4c framework, the adoption of high-level fully relativistic DIP-EOMCC methodologies has been comparatively slower. The previous 4c-DIP-EOMCC approaches of refs 26–28 were limited to the DIP-EOMCCSD­(3*h*-1*p*) level,^3–9^ which treats 2*h* and 3*h*-1*p* excitations on top of CC with singles and doubles (CCSD).^29–32^ More recently, the high-level DIP-EOMCCSDT­(4*h*-2*p*) approach was extended to the two-component relativistic regime in ref 33, however, due to excessive CPU and memory requirements, the resulting DIP-EOMCCSDT­(4*h*-2*p*) calculations were limited to triple-ζ-quality basis sets. Thus, a major aim of the present study is to develop 4c-DIP-EOMCC approaches that incorporate up to 4*h*-2*p* correlations and three-body clusters in a robust and practical fashion, and which can be applied to systems described with larger quadruple-ζ (QZ) basis sets. To accomplish this task, we follow two strategies for reducing computational costs. First, we simplify the treatment of 4*h*-2*p* excitations in the DIP-EOMCCSD­(T)­(a)­(4*h*-2*p*) method to obtain a new approach abbreviated as DIP-EOMCCSD­(T)­(*ã*)­(4*h*-2*p*), which can be performed using computational steps that scale as $N^{7}$ with the system size. In addition, we adopt the frozen natural spinor (FNS) technique,^34,35^ as implemented in ref 36, to reduce the numbers of virtual spinors entering the 4c-DIP-EOMCC calculations in a controlled and systematic fashion. The resulting FNS-based 4c-DIP-EOMCCSD­(T)­(*ã*)­(4*h*-2*p*) calculations are applied to obtain the DIPs of the series of inert gas atoms from argon to radon as well as the vertical DIPs in Cl_2_, Br_2_, HBr, and HI using uncontracted Dyall-type basis sets of up to QZ quality containing as many as 764 orbitals. This allows us to carry out complete basis set (CBS) extrapolations in order to make more meaningful comparisons with the existing experimental data.

## 2 Theory

### 2.1 Treatment of Relativistic Effects

Relativistic interactions in quantum chemistry are typically described using a 4c Dirac–Coulomb (DC) Hamiltonian,^37^ which is given (in atomic units) for an *N*-electron system with *M* clamped nuclei by

H^DC=∑i=1N[cα→i·p→i+βim0c2+∑A=1MV^iA]+∑j>i=1N1rijI^4, 1

where *c* is the speed of light, *m*_0_ is the rest mass of an electron, and ${\hat{V}}_{iA}$ is the electrostatic attraction between electron *i* and nucleus *A*. As usual, ${\vec{p}}_{i}$ is the momentum of the *i*th electron and ${\vec{\alpha }}_{i}$ and β_*i*_ denote the Dirac matrices associated with electron *i*. The operator I^4 in eq 1 is a 4 × 4 identity matrix. A zeroth-order description of the many-electron system is obtained by solving the Dirac–Hartree–Fock (DHF) mean-field equations. The matrix DHF equations are expressed as

(V^+J^−K^c(σ·p^)−K^c(σ·p^)−K^V^−2m0c2+J^−K^)(ϕLϕS)=E(ϕLϕS), 2

where ϕ^L^ (ϕ^S^) refers to the large (small) component of the 4c spinor ψ. The operators $\hat{V}$ , $\hat{J}$ , and $\hat{K}$ in eq 2 denote the electron–nuclear, Coulomb, and exchange potentials, respectively. While the DC Hamiltonian serves as the natural starting point for relativistic quantum chemical calculations, one can also consider the Gaunt (DCG) and Breit (DCB) corrections to the DC Hamiltonian, which are given by

$${\hat{H}}^{\mathrm{DCG}}=\sum_{i=1}^{N}\left[c{\vec{\alpha }}_{i}.{\vec{p}}_{i}+\beta_{i}m_{0}c^{2}+\sum_{A=1}^{M}{\hat{V}}_{iA}\right]+\sum_{j>i=1}^{N}\left(\frac{1}{r_{ij}}+G_{ij}\right){\hat{I}}_{4}$$ 3

and

H^DCB=∑i=1N[cα→i.p→i+βim0c2+∑A=1MV^iA]+∑j>i=1N(1rij+Bij)I^4, 4

respectively, where

$$G_{ij}=-\frac{\alpha_{i}\cdot \alpha_{j}}{2r_{ij}}$$ 5

and

$$B_{ij}=-\frac{1}{2r_{ij}}\left[\alpha_{i}\cdot \alpha_{j}+\frac{\left(\alpha_{i}\times r_{ij})\cdot (\alpha_{j}\times r_{ij}\right)}{r_{ij}^{2}}\right].$$ 6

After obtaining the DHF mean-field state via eq 2 using either the DC, DCG, or DCB Hamiltonian, the missing many-electron correlation effects can be incorporated using the no-pair approximation.^38^ We note that when using the DCG or DCB Hamiltonians, the Gaunt or Breit corrections are only included in the DHF step and are neglected in the subsequent integral transformation.

### 2.2 Overview of the Double Ionization Potential Equation-of-Motion Coupled-Cluster Method

In order to treat many-electron correlation effects on top of the DHF mean-field solution, we rely on the hierarchy of approximations based on the CC theory alongside its DIP-EOMCC extension to doubly ionized states. In the single-reference CC theory,^39–43^ the ground-state many-body wave function for an *N*-electron system is described using the exponential wave function ansatz^44,45^

|Ψ0(N)⟩=eT^|Φ⟩, 7

where |Φ⟩ is the DHF determinant, which serves as a Fermi vacuum, and $\hat{T}$ is the cluster operator,

T^=∑n=1MTT^n, 8

where the *n*-body component of $\hat{T}$ is

T^n=∑i1<···<ina1<···<anta1···ani1···ina^a1···a^ana^in···a^i1. 9

As usual, indices *i*_1_, *i*_2_, ··· (*a*_1_, *a*_2_, ···) denote the spinors that are occupied (unoccupied) in |Φ⟩ and *â* ^*p*^ (*â*_*p*_) represents the Fermionic creation (annihilation) operator associated with the spinor |*p*⟩. The value *M*_*T*_ in eq 8 controls the truncation in $\hat{T}$ , which gives rise to the conventional hierarchy of CC approximations. For example, *M*_*T*_ = 2 defines the basic CCSD method, in which $\hat{T}$ = $\hat{T}$ _1_ + $\hat{T}$ _2_, while *M*_*T*_ = 3 yields the higher-level CCSDT approach with $\hat{T}$ = $\hat{T}$ _1_ + $\hat{T}$ _2_ + $\hat{T}$ _3_, and so on. For a given truncation *M*_*T*_, the amplitudes characterizing the cluster operator $\hat{T}$ are determined by solving the projective conditions,

$$\langle \Phi_{i_{1}\cdot \cdot \cdot i_{n}}^{a_{1}\cdot \cdot \cdot a_{n}}|\overset{-}{H}|\Phi \rangle =0,\;i_{1}<\cdot \cdot \cdot <i_{n},\;a_{1}<\cdot \cdot \cdot <a_{n},$$ 10

for *n* = 1, ···, *M*_*T*_, where

$$\overset{-}{H}=e^{-\hat{T}}\hat{H}e^{\hat{T}}$$ 11

is the corresponding similarity-transformed Hamiltonian. After solving eq 10 to obtain the cluster amplitudes *t*_*a*_1_···*a*_*n*__^*i*_1_···*i*_*n*_^, *n* = 1, ···, *M*_*T*_, the ground-state energy is obtained *a posteriori* as

$$E_{0}=\langle \Phi |\overset{-}{H}|\Phi \rangle .$$ 12

It has been well established over the course of many studies employing nonrelativistic (NR) Hamiltonians (cf. refs 46 and 47 for representative examples) that the higher-level CC approximations, like CCSDT (*M*_*T*_ = 3) and CCSDTQ (*M*_*T*_ = 4),^48–51^ are capable of recovering a highly accurate treatment of the many-electron correlation effects relative to the exact, full CI, solution in most chemically relevant problems, including noncovalent interactions, bond dissociations, and open shells, like radicals and biradicals. The same is true when examining the convergence of the CC hierarchy applied to relativistic Hamiltonians.^52,53^

Doubly ionized states can be treated within the CC framework using the DIP-EOMCC methodology in which the ground (μ = 0) and excited (μ > 0) states of the (*N* – 2)-electron target system are described as

|Ψμ(N−2)⟩=R^μ(−2)|Ψ0(N)⟩, 13

where the doubly ionizing operator

$${\hat{R}}_{\mu }^{\left(-2\right)}=\sum_{n=0}^{M_{R}}{\hat{R}}_{\mu ,(n+2)h‐np}$$ 14

consists of many-body components

R^μ,(n+2)h‐np=∑i<j<k1<···<knc1<···<cnrc1···cnijk1···kn(μ)a^c1···a^cna^kn···a^k1a^ja^i 15

that remove two electrons from the *N*-electron ground-state wave function |Ψ_0_^(*N*)^⟩ via (*n* + 2)*h*-*np* excitations. The truncation parameter *M*_*R*_ in eq 14 determines the maximum level of (*n* + 2)*h*-*np* excitations included in $\hat{R}$ _μ_^(−2)^. By varying the values of *M*_*T*_ and *M*_*R*_, we obtain the standard hierarchy of DIP-EOMCC approximations.

For example, DIP-EOMCC methods based on a CCSD description of the *N*-electron system (*M*_*T*_ = 2) include the DIP-EOMCCSD­(3*h*-1*p*) and DIP-EOMCCSD­(4*h*-2*p*)^8,9^ approaches, which include up to the 3*h*-1*p* (*M*_*R*_ = 1) and 4*h*-2*p* (*M*_*R*_ = 2) excitations in the (*N* – 2)-electron species, respectively. One can also consider DIP-EOMCC methods based on the more accurate *N*-electron CCSDT state (*M*_*T*_ = 3), such as the DI-EOMCCSDT scheme,^5^ which treats the 2*h* and 3*h*-1*p* excitations (*M*_*R*_ = 1) on top of CCSDT, and the recently introduced DIP-EOMCCSDT­(4*h*-2*p*) approach corresponding to *M*_*T*_ = 3 and *M*_*R*_ = 2 that provides a full treatment of both 4*h*-2*p* and $\hat{T}$ _3_ correlations. While all of the aforementioned DIP-EOMCC methods are convenient tools for determining DIPs in many-electron systems, the study in ref 20 emphasizes that obtaining highly accurate DIPs requires that one balances the correlations due to (*n* + 2)*h*-*np* excitations in the (*N* – 2)-electron target states with the CC treatment of the underlying *N*-electron system. In particular, when 4*h*-2*p* excitations are included in the $\hat{R}$ _μ_^(−2)^ operator, which is often necessary for obtaining accurate energetics,^8,9,20^ one must also account for $\hat{T}$ _3_ correlations in the *N*-electron ground state.

Given a particular truncation of the $\hat{R}$ _μ_^(−2)^ and $\hat{T}$ operators, a typical DIP-EOMCC calculation proceeds by solving the matrix eigenvalue problem, which for *M*_*R*_ ≤ *M*_*T*_ (a condition required to obtain size-intensive DIPs^8,9,54^ ) is given by

[H−open,R^μ(−2)]|Φ⟩=ωμ(N−2)R^μ(−2)|Φ⟩, 16

where *H̅*_open_ refers to the diagrams in *H̅* that contain external Fermion lines. The eigenvalues ω_μ_^(*N*–2)^ obtained by solving eq 16 are the DIPs corresponding to vertical transitions between the *N*-electron ground state |Ψ_0_^(*N*)^⟩ and the ground (μ = 0) and excited (μ > 0) states of the (*N* – 2)-electron system, |Ψ_μ_^(*N*–2)^⟩, while the corresponding right eigenvectors provide the excitation amplitudes *r*_*c*_1_···*c*_*n*__^*ijk*_1_···*k*_*n*_^(μ), for *n* = 0, ···, *M*_*R*_, characterizing the $\hat{R}$ _μ_^(−2)^ operator. Thus, the post-DHF steps of a 4c-DIP-EOMCC calculation consist of solving eq 10 to obtain the truncated form of the cluster operator $\hat{T}$ and energy *E*_0_ (eq 12) characterizing the *N*-electron ground state |Ψ_0_^(*N*)^⟩ and diagonalizing the corresponding similarity-transformed Hamiltonian *H̅* (eq 11) in the appropriate (*N* – 2)-electron subspace of the Fock space associated with the content of R^μ(−2) following eq 16.

In the full DIP-EOMCCSDT­(4*h*-2*p*) method, which is the highest level of DIP-EOMCC theory considered in this work, the cluster operator is given by $\hat{T}$ = $\hat{T}$ _1_ + $\hat{T}$ _2_ + $\hat{T}$ _3_ and the doubly ionizing operator is truncated at the 4*h*-2*p* level to yield $\hat{R}$ _μ_^(−2)^ = $\hat{R}$ _μ,2*h*_ + $\hat{R}$ _μ,3*h*‑1*p*_ + $\hat{R}$ _μ,4*h*‑2*p*_. As a result, the DIP-EOMCCSDT­(4*h*-2*p*) calculation involves computational steps that scale as *n*_*o*_^3^ *n*_*u*_^5^, corresponding to the preliminary CCSDT calculation for the underlying *N*-electron system, followed by the diagonalization of the Hamiltonian in the subspace of the Fock space associated with $\hat{R}$ _μ_^(−2)^, which scales as *n*_*o*_^4^ *n*_*u*_^4^, where *n*_*o*_ (*n*_*u*_) denotes the number of occupied (unoccupied) spinors in |Φ⟩. Both of these steps scale as $N^{8}$ with the system size N , however, the preliminary CCSDT step is significantly more expensive due to the *n*_*u*_^5^ scaling with the number of unoccupied spinors.

The DIP-EOMCCSD­(T)­(a)­(4*h*-2*p*) approximation to DIP-EOMCCSDT­(4*h*-2*p*) introduced in ref 20 provides a solution to this issue by replacing the CCSDT step by its much less expensive CCSD analog, which involves computational steps that scale as *n*_*o*_^2^ *n*_*u*_^4^. In DIP-EOMCCSD­(T)­(a)­(4*h*-2*p*), the $\hat{T}$ _3_ cluster is approximated according to perturbation theory,

T^3[2]=D^3[V^N,T^2], 17

where $\hat{T}$ _2_ in eq 17 refers to the two-body component of $\hat{T}$ obtained from the CCSD calculation, we have adopted the Mo̷ller–Plesset (MP) partitioning of the electronic Hamiltonian, $\hat{H}$ _*N*_ = $\hat{H}$ – ⟨Φ| $\hat{H}$ |Φ⟩ = $\hat{F}$ _*N*_ + ${\hat{V}}_{N}$ , with $\hat{F}$ _*N*_ and ${\hat{V}}_{N}$ representing the usual Fock and fluctuation operators resulting from normal ordering of the Hamiltonian with respect to |Φ⟩, and $\hat{D}$ _3_ generates the three-body MP energy denominator. The one- and two-body components of $\hat{T}$ determined in the *N*-electron CCSD calculation are then noniteratively corrected for the $\hat{T}$ _3_ effects via

$${\hat{T}}_{1}^{\prime }={\hat{T}}_{1}+{\hat{D}}_{1}[{\hat{V}}_{N},{\hat{T}}_{3}^{\left[2\right]}]$$ 18

and

T^2′=T^2+D^2[H^N,T^3[2]], 19

where $\hat{D}$ _1_ and $\hat{D}$ _2_ generate the one- and two-body MP energy denominators. Using $\hat{T}$ _1_ *′*, $\hat{T}$ _2_ *′*, and $\hat{T}$ _3_^[2]^, the CCSD­(T)­(a) similarity-transformed Hamiltonian,^25^ represented in the (*N* – 2)-electron subspace of the Fock space spanned by the 2*h*, 3*h*-1*p*, and 4*h*-2*p* excitations as

H−[CCSD(T)(a)]=(H−2h,2h′H−2h,3h‐1p′H−2h,4h‐2p′H−3h‐1p,2h′+[V^N,T^3[2]]H̅3h‐1p,3h‐1p′H−3h‐1p,4h‐2p′H−4h‐2p,2h′+[F^N+V^N,T^3[2]]H−4h‐2p,3h‐1p′+[V^N,T^3[2]]H−4h‐2p,4h‐2p′), 20

where *H̅* *′* = e−T^′1−T^′2 H^eT^′1+T^′2 , can be constructed and diagonalized to obtain the vertical DIPs and excitation amplitudes characterizing the $\hat{R}$ _μ_^(−2)^ operator. In this way, the DIP-EOMCCSD­(T)­(a)­(4*h*-2*p*) approximation avoids the expensive *N*-electron CCSDT calculation and reduces the *n*_*o*_^3^ *n*_*u*_^5^ costs characterizing its DIP-EOMCCSDT­(4*h*-2*p*) parent to *n*_*o*_^4^ *n*_*u*_^4^. As shown in ref 20, the DIPs obtained using the DIP-EOMCCSD­(T)­(a)­(4*h*-2*p*) approach are very close to those computed with DIP-EOMCCSDT­(4*h*-2*p*) when examining the vertical transitions in closed-shell molecules near their equilibrium ground-state structures.

In the 4c framework adopted in the present study, the *n*_*o*_^4^ *n*_*u*_^4^ steps entering the DIP-EOMCCSD­(T)­(a)­(4*h*-2*p*) calculations proved to be very computationally demanding, especially for larger QZ basis sets, so we invoked three additional approximations motivated by practicality, resulting in the method abbreviated as DIP-EOMCCSD­(T)­(*ã*)­(4*h*-2*p*). In DIP-EOMCCSD­(T)­(*ã*)­(4*h*-2*p*), the CCSD­(T)­(a) similarity-transformed Hamiltonian is further simplified by (i) removing all contributions to 3-body components of *H̅* ^[CCSD(T)(a)]^ arising from contractions with $\hat{T}$ _3_ clusters, (ii) neglecting all $\hat{T}$ _3_ contributions in the projections corresponding onto 4*h*-2*p* determinants, and (iii) assuming that *H̅* ^[CCSD(T)(a)]^ is quasi-diagonal in the 4*h*-2*p* sector of the Fock space, allowing us to replace *H̅* *′*_4*h*‑2*p*,4*h*‑2*p*_ by its zeroth-order, MP-like, counterpart. The programmable expressions for the left-hand sides of the DIP-EOMCCSD­(T)­(*ã*)­(4*h*-2*p*) equations corresponding to projections onto 2*h* (|Φ_*ij*_⟩), 3*h*-1*p* (|Φ_*ijk*_^*c*^⟩), and 4*h*-2*p* (|Φ_*ijkl*_^*cd*^⟩) determinants, as implemented in this work, are

⟨Φij|(H−N,open(CCSD(T)(a~))R^μ(−2))C|Φ⟩=Aij[−h−mirmj(μ)+14h−mnijrmn(μ)+12h−mereijm(μ)−12h−mnifrfmjn(μ)+18h−mnefrefijmn(μ)], 21

⟨Φijkc|(H−N,open(CCSD(T)(a~))R^μ(−2))C|Φ⟩=Aijk[12I′ie(μ)tecjk−12h−cmkirmj(μ)+16h−cereijk(μ)−12h−mkrcijm(μ)+14h−mnijrcmnk(μ)+12h−cmkereijm(μ)+16h−merceijkm(μ)−14h−mnkfrcfijmn(μ)], 22

and

⟨Φijklcd|(H−N,open(CCSD(T)(a~))R^μ(−2))C|Φ⟩=AijklAcd[112h−dclereijk(μ)−14h−dmlkrcijm(μ)−112Imijk(μ)tcdml+14Icije(μ)tedkl+124fderceijkl(μ)−112fmircdmjkl(μ)], 23

where the index antisymmetrizers in eqs 21–23 are

$$A^{pq}=A_{pq}=1-(pq),$$

$$A^{pqr}=A^{p/qr}A^{qr},$$

and

$$A^{pqrs}=A^{p/qrs}A^{qrs},$$

with the partial antisymmetrizers defined as

$$A^{p/qr}=1-\left(pq\right)-\left(pr\right)$$

and

$$A^{p/qrs}=1-\left(pq\right)-\left(pr\right)-\left(ps\right).$$

The quantities *f*_*p*_^*q*^ in eq 23 denote the standard one-electron Fock matrix, while the expressions for the remaining one-body (*h̅*_*p*_^*q*^) and two-body (*h̅*_*pq*_^*rs*^) components of the similarity-transformed Hamiltonian as well as additional intermediates entering eqs 21–23 are provided in the Supporting Information. The C subscript indicates connected product.

For the recommended DIP-EOMCCSD­(T)­(*ã*)­(4*h*-2*p*) method, the most computationally demanding step is the construction of the $\hat{T}$ _3_ cluster amplitudes, which scales as *n*_*o*_^3^ *n*_*u*_^4^, whereas the dominant term in the EOMCC matrix–vector product scales as *n*_*o*_^4^ *n*_*u*_^3^. Since *n*_*u*_ > *n*_*o*_ in typical applications, the overall computational cost is governed by the *n*_*o*_^3^ *n*_*u*_^4^ scaling. The FNS approximation reduces the number of virtual spinors. For a truncation of *f* % of the virtual space, the number of active virtual spinors is given by

$$n_{u}^{\mathrm{active}}=\left(1-\frac{f}{100}\right)n_{u}.$$ 24

Consequently, the computational cost of the ground-state CC and DIP-EOMCC calculations is reduced to ${\left(1-\frac{f}{100}\right)}^{4}$ and ${\left(1-\frac{f}{100}\right)}^{3}$ times that of the corresponding calculations performed with the full virtual space, respectively.

### 2.3 Frozen Natural Spinor Technique

The natural spinors^34^ are the relativistic analogs of the NR natural orbitals, introduced by Löwdin.^55^ Natural spinors are obtained as the eigenfunctions of the relativistic correlated one-body reduced density matrix.^56^ Among the various schemes for obtaining natural spinors,^34,57,58^ we have chosen the standard MP2-based natural spinors,^34^ which are obtained by rotating the set of unoccupied DHF spinors using the eigenvectors of the virtual–virtual block of the one-body reduced density matrix computed at the MP2 level.

After constructing the MP2 one-body reduced density matrix and diagonalizing it, the eigenvalues are occupancies of the corresponding virtual natural spinors (eigenvectors),

DV=Vn. 25

Sorting the natural spinors based on their occupancies in a decreasing order leads to a gradual hierarchy of their contribution to the correlation. One can set up a predefined threshold (*n*_thresh_) to truncate them, where only the natural spinors with occupancies larger than *n*_thresh_ are considered and the rest of them are dropped off in the following calculations. Truncation of the virtual space can be accomplished by multiplying the natural spinor transformation matrix *V* by a thresholding matrix τ according to

$$\overset{\sim }{V}=V\tau ,$$ 26

where

$$\begin{array}{ll} \tau_{ij}=\delta_{ij} & \forall n_{i}>n_{\mathrm{thresh}},\\ \tau_{ij}=0 & \forall n_{i}\leq n_{\mathrm{thresh}}. \end{array}$$ 27

The virtual–virtual block of the Fock matrix is then transformed into the natural spinor basis according to

$$\overset{\sim }{F}={\overset{\sim }{V}}^{†}F\overset{\sim }{V}.$$ 28

Here, a tilde is used to denote a matrix expressed in the truncated basis. Diagonalizing $\overset{\sim }{F}$ leads to natural spinor energies (ϵ̃) as eigenvalues and eigenvectors $\left(\overset{\sim }{Z}\right)$ , which are used to semicanonicalize the new basis,

$$\overset{\sim }{F}\overset{\sim }{Z}=\overset{\sim }{Z}\overset{\sim }{\varepsilon }.$$ 29

In practice, the transformation between the original virtual molecular spinors and the truncated virtual natural spinor basis is

$$\overset{\sim }{B}=\overset{\sim }{V}\overset{\sim }{Z}.$$ 30

The atomic spinor integrals can be directly converted to the truncated natural spinor basis by the following transformation matrices:

$${\overset{\sim }{U}}_{\mathrm{occ}}=U_{\mathrm{occ}},$$ 31

$${\overset{\sim }{U}}_{\mathrm{vir}}=U_{\mathrm{vir}}\overset{\sim }{V}\overset{\sim }{Z}=U_{\mathrm{vir}}\overset{\sim }{B},$$ 32

where *U* represents the transformation matrix between the atomic spinor basis and the molecular spinor (DHF) basis. This approach is called FNS,^34,35,57,59,60^ as the occupied sector is kept frozen at the DHF level of theory.

## 3 Computational Details

The 4c DIP-EOMCCSDT­(4*h*-2*p*), DIP-EOMCCSDT­(a)­(4*h*-2*p*), and DIP-EOMCCSD­(T)­(*ã*)­(4*h*-2*p*) methods along with their FNS-truncated counterparts have been implemented in BAGH,^61^ our in-house quantum chemistry software designed for advanced computational wave function-based calculations (in doing so, we benefited from our previously developed DIP-EOMCC routines reported in ref 20). BAGH is mainly written in Python, with the bottleneck parts being optimized using Cython and Fortran. It is currently compatible with four interfaces: PySCF,^62–64^ Socutils,^65^ GAMESS-US,^66^ and DIRAC.^67^ Among other capabilities, the BAGH software can perform DIP-EOMCC calculations using both NR and various relativistic Hamiltonians.

The uncontracted dyall.av*n*z (*n* = 2,3,4) basis sets^18,19^ have been used for the valence DIP energy calculations of inert gas atoms (Ar–Rn) as well as the Cl_2_, Br_2_, HBr, and HI molecules. All diatomic molecules were described using their experimental bond lengths obtained from ref 68. The lowest-energy occupied orbitals corresponding to the chemical cores of the elements were frozen in all post-HF steps of the DIP-EOMCC calculations. The effect of including diffuse functions on the resulting DIPs has been studied by augmenting the dyall.av4z basis set with single, double, and triple sets of diffuse functions. The augmented basis sets were generated using the DIRAC software package.^67^

The calculated DIP values were extrapolated to the complete-basis-set (CBS) limit using the dyall.av*n*z basis sets (*n* = 2–4). The total energies of the ground state and the doubly ionized states were extrapolated independently, and the final DIP energies were obtained as the differences between the two extrapolated energies.

To the best of our knowledge, four commonly used formulations for CBS extrapolation have been reported in the literature. In the first approach, the SCF contribution is extrapolated using Feller’s formulation,^69^ combined with the scheme of either Helgaker et al.^70^ or Lesiuk and Jeziorski^71^ to extrapolate the correlation energy. A third formulation, proposed by Martin,^72^ applies extrapolation directly to the total energy. Finally, there exists Peterson et al.’s^73^ CBS extrapolation scheme, which is based on total energies. The 4c-FNS-DIP-EOMCCSD­(3*h*-1*p*) results for molecules extrapolated to the CBS limit using all four schemes are provided in the Supporting Information, and the four extrapolation methods yield mutually consistent results (See Table S3). In this work, the CBS extrapolation is performed using the mixed exponential formula of Peterson et al.,^73^

$$E^{x}=E^{\infty }+Ae^{-(x-1)}+Be^{-(x-1{)}^{2}},$$ 33

where *A* and *B* are parameters and *E* ^*x*^ and *E* ^*∞*^ are the total energies for a particular basis (*x*) and at the CBS limit, respectively.

After performing a DHF calculation, we construct two-electron integrals of the ⟨*OO*||*VV*⟩ type in the canonical spinor basis in order to perform the MP2 calculation and compute the corresponding virtual–virtual block of the one-body reduced density matrix. The virtual space is then truncated according to a predefined FNS threshold *n*_thresh_ using the recipe described in Section 2.3. All one- and two-electron integrals are computed and stored in the truncated natural spinor basis with the help of the transformation matrices given by eqs 31 and 32, which allow us to move from the atomic spinor to FNS space in a computationally efficient manner. The CCSD calculation is performed in the FNS basis and the amplitudes are noniteratively corrected for $\hat{T}$ _3_ effects using eqs 18 and 19. In the DIP-EOMCCSD­(T)­(*ã*)­(4*h*-2*p*) calculations, the $\hat{T}$ _3_ contributions to the *h̅*_*am*_^*ij*^ component of *H̅* entering eqs 22 and 23 are computed prior to solving the DIP-EOMCC eigenvalue problem. A schematic diagram of the algorithm for the FNS-DIP-EOMCCSD­(T)­(*ã*)­(4*h*-2*p*) approach is provided in Figure 1. The FNS-DIP-EOMCCSDT­(4*h*-2*p*) and DIP-EOMCCSD­(T)­(a)­(4*h*-2*p*) calculations follow the same algorithm defined in ref 20, after the one- and two-electron integrals are generated in FNS basis.

**Figure 1:**
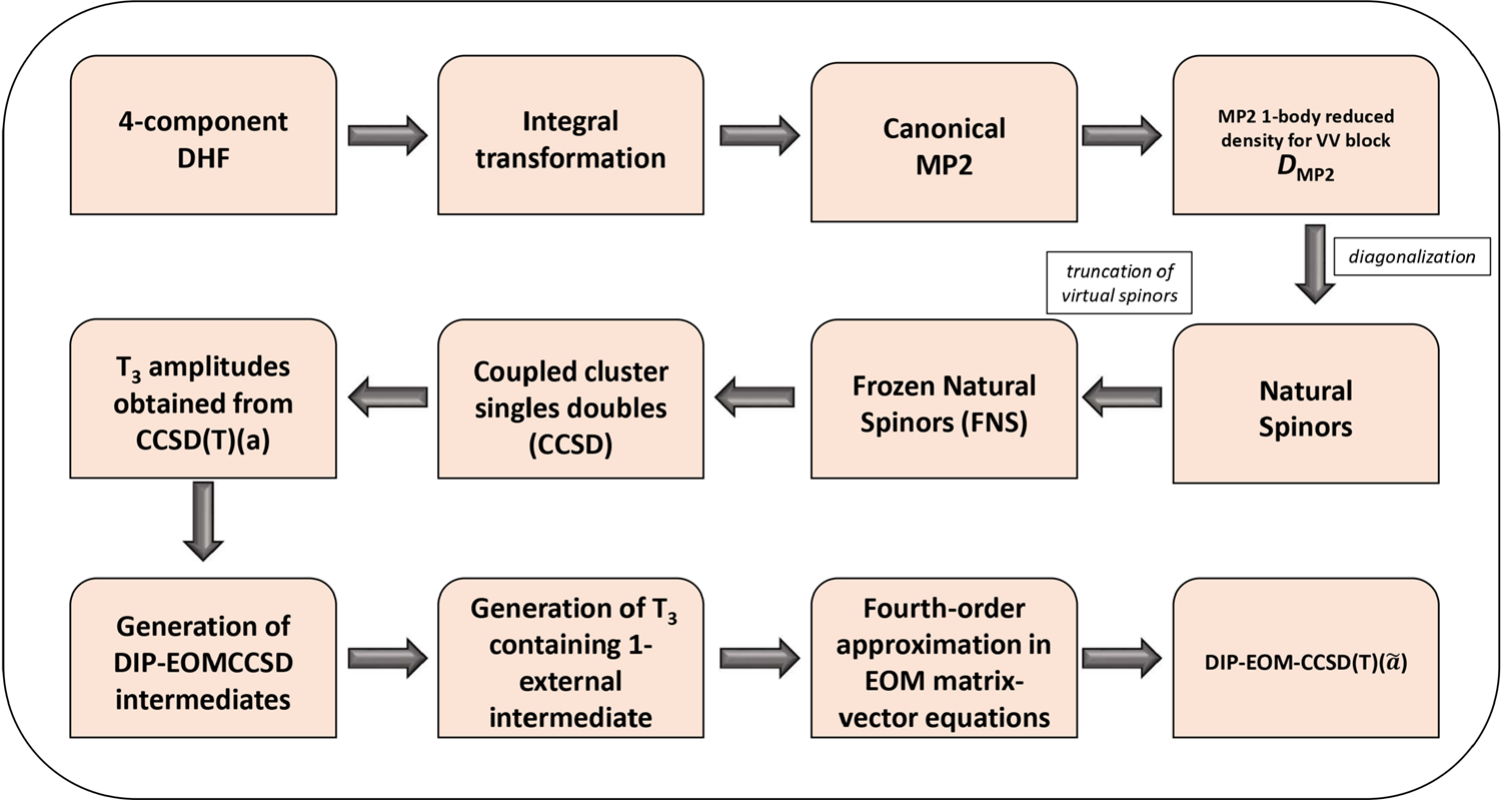
Schematic diagram of the algorithm of the FNS-DIP-EOMCCSD­(T)­(*ã*)­(4*h*-2*p*) method.

## 4 Results and Discussion

### 4.1 Choice of FNS Threshold

The main idea behind the FNS approximation is to reduce the size of the virtual space in correlated relativistic calculations. The truncation in the canonical spinor basis leads to a significant loss of accuracy due to the contribution of high-lying virtual spinors to the energy. On the other hand, the virtual spinors in the natural spinor basis are arranged according to their occupancy, which roughly tracks their contribution to the correlation energy. For the case of DIP, where its zeroth-order description does not involve any virtual spinors, the FNS basis can give a compact description for the DIP-EOMCCSD­(3*h*-1*p*) and DIP-EOMCCSD­(4*h*-2*p*) calculations similar to that observed for the FNS-IP-EOMCCSD method.^36^ To illustrate this point, we present the convergence of the lowest valence DIP for Cl_2_ with respect to the size of the truncated virtual space in Figure 2a using 4c-FNS-DIP-EOMCCSD­(3*h*-1*p*). It can be seen that the DIP values converge more quickly in the FNS basis and the results approach their canonical counterparts using just 40% of the total virtual space. In the canonical basis, one needs to include at least 60% of the virtual space to achieve a similar level of accuracy. However, the FNS threshold is a better truncation criterion than the size of the virtual space. Figure 2b presents the convergence of absolute error characterizing the lowest-lying DIP of Cl_2_ computed with 4c-FNS-DIP-EOMCCSD­(3*h*-1*p*) as a function of the FNS threshold. The calculation is performed in dyall.av3z basis set and the 4c-DIP-EOMCCSD­(3*h*-1*p*) values in the untruncated canonical basis have been taken as the reference. From Figure 2b, the truncation error in the DIP for the *X* ^3^Σ^–^ state of (Cl_2_)^2+^ is less than 0.1 eV using an FNS threshold of 10^–4^. With an FNS threshold of 10^–5^, the DIP obtained using the FNS-based DIP-EOMCCSD­(3*h*-1*p*) approach is virtually identical to its untruncated counterpart. However, considering the fact that the FNS threshold is directly related to the number of virtual spinors that will be considered for correlation calculations, taking 10^–5^ as the FNS threshold for calculations may be a costly choice. The FNS threshold of 10^–4.5^ seems to be a good compromise between cost and accuracy as the truncation error characterizing the DIP for the *X* ^3^Σ^–^ state of (Cl_2_)^2+^ is as little as 0.01 eV. Based on these observations, we adopt an FNS occupation threshold of 10^–4.5^ for all calculations, unless otherwise mentioned.

**Figure 2:**
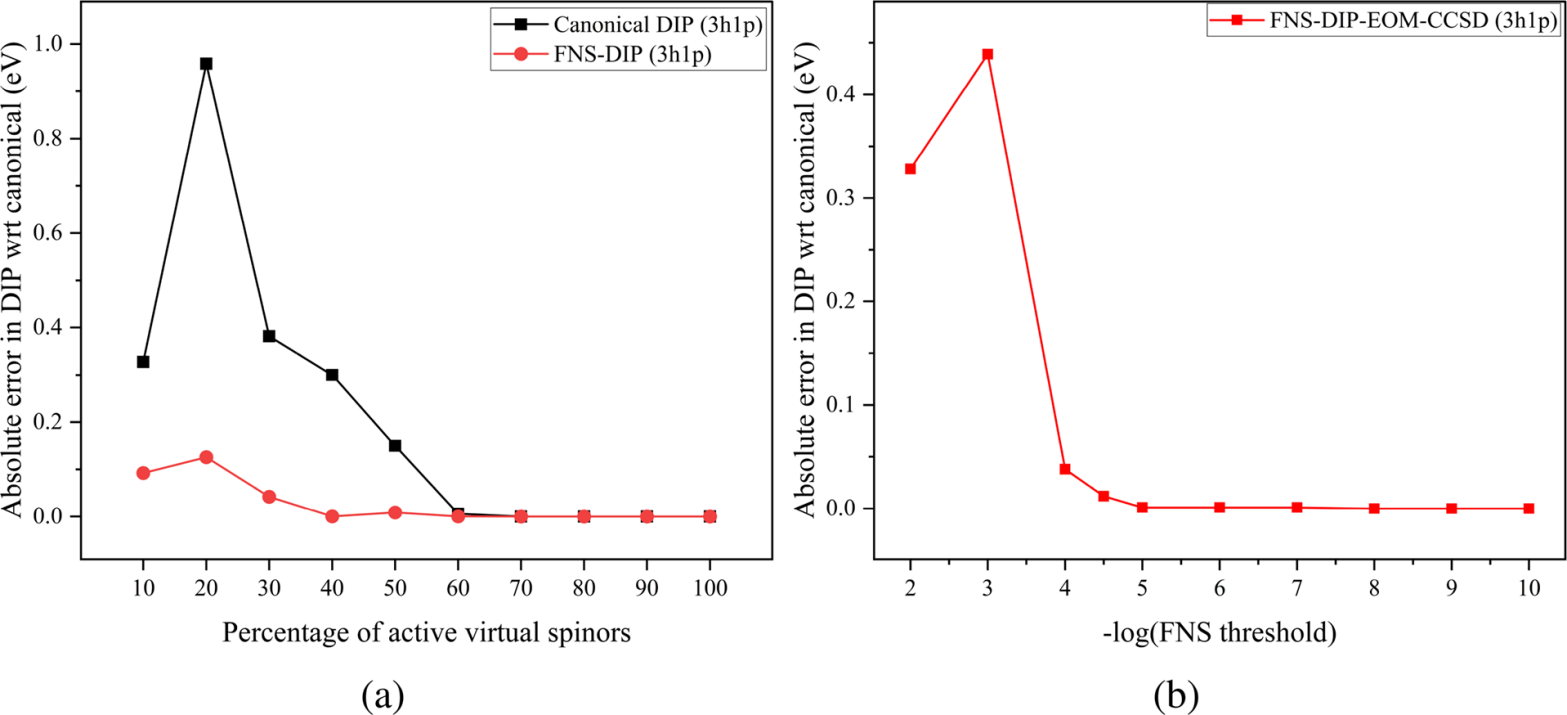
Comparison of absolute error of DIP energies (in eV) characterizing the *X* ^3^Σ^–^ state of Cl_2_ for the FNS version of 4c-DIP-EOMCCSD­(3*h*-1*p*) with respect to their respective canonical analogues calculated using dyall.av3z basis set (a) across the percentage of active virtual spinors and (b) across different truncation thresholds.

### 4.2 Basis Set Convergence

To analyze the effect of basis set, we have compared the DIPs corresponding to the *X* ^3^Σ^–^, *a* ^1^Δ, *b* ^1^Σ^+^, and *c* ^1^Σ^–^ states of (Cl_2_)^2+^, as obtained with DIP-EOMCCSD­(3*h*-1*p*) using the dyall.av*n*z (*n* = 2,3,4) hierarchy, with their experimentally determined counterparts in Table 1. Furthermore, in order to investigate the effect of diffuse functions on the calculated DIP values, the results obtained with dyall.av4z have been augmented with single, double, and triple sets of diffuse functions. It can be seen that the DIP values increase with the basis set cardinality number. For example, the DIPs characterizing the valence states of (Cl_2_)^2+^ grow by roughly 0.3 eV when the basis set is increased from dyall.av2z to dyall.av3z. Slightly smaller changes of ∼0.2 eV are observed when increasing the basis from dyall.av3z to dyall.av4z. As shown in Table 1, the DIP values obtained with dyall.av4z increase by 0.1–0.2 eV when CBS extrapolations are employed. Thus, even the dyall.av4z basis results in significant basis set errors.

**Table 1:** Basis Set Convergence of 4c-FNS-DIP-EOMCCSD­(3*h*-1*p*) DIP Values (in eV) of Cl_2_ Molecule in Different Dyall Basis Sets

state	dyall.av2z	dyall.av3z	dyall.av4z	s-aug-dyall.av4z	d-aug-dyall.av4z	t-aug-dyall.av4z	CBS^a^	expt.^74^
*X* ^3^Σ^–^	31.03	31.39	31.58	31.59	31.59	31.59	31.72	31.13
*a* ^1^Δ	31.58	31.91	32.09	32.09	32.09	32.09	32.20	31.74
*b* ^1^Σ^+^	31.94	32.30	32.48	32.48	32.48	32.48	32.70	32.12
*c* ^1^Σ^–^	32.98	33.32	33.51	33.51	33.51	33.51	33.63	32.97

a The CBS value is calculated by extrapolating dyall.av*n*z (*n* = 2,3,4) basis sets.

In contrast, the inclusion of diffuse functions in the basis set provides hardly any effect on the resulting DIP values. As shown in Table 1, the DIP values computed with 4c-DIP-EOMCCSD­(3*h*-1*p*) using the dyall.av3z basis show the best agreement with experiment. However, this agreement vanishes when a larger dyall.av4z basis is employed. One needs to use a higher-level treatment of many-electron correlation effects to obtain an accurate and systematic behavior of the DIP values.

### 4.3 Effect of the Inclusion of Higher Excitations

In order to investigate the effect of the inclusion of higher-rank excitation and doubly ionizing operators, the DIPs characterizing the *X* ^3^Σ^–^, *a* ^1^Δ, *b* ^1^Σ^+^, and *c* ^1^Σ^–^ states of (Cl_2_)^2+^ computed with different levels of 4c-DIP-EOMCC approximation have been presented in Table 2. The FNS-DIP-EOMCCSD­(3*h*-1*p*) results are calculated in dyall.av3z and dyall.av4z bases. The results obtained from the dyall.av3z basis have been shown for the sake of comparison with the previously reported canonical values by Pal and co-workers.^27^ It can be seen that at the FNS-DIP-EOMCCSD­(3*h*-1*p*)/dyall.av3z level of theory, the resulting DIPs show good agreement with their experimental counterparts. The FNS-DIP-EOMCCSD­(3*h*-1*p*)/dyall.av3z DIP values are also nearly identical to the canonical results reported by Pal and co-workers^27^ using the same level of theory. However, the DIPs computed with FNS-DIP-EOMCCSD­(3*h*-1*p*)/dyall.av4z are significantly larger than experiment. The inclusion of the 4*h*-2*p* excitations on top of CCSD leads to an underestimation of the DIP values. This behavior arises because the inclusion of the 4*h*-2*p* excitation space within the DIP-EOMCCSD­(4*h*-2*p*) framework improves the description of the (*N* – 2)-electron target states. However, the resulting DIPs tend to be underestimated, as the target states are treated at a higher level of correlation than the *N*-electron reference state, thereby artificially contracting the energy gap. Incorporating triples corrections for the *N*-electron ground state, together with the 4*h*-2*p* excitation manifold for the (*N* – 2)-electron target states, restores a balanced level of correlation between the two sectors and yields highly accurate DIP values. This imbalance and its resolution have been discussed in detail in our previous work on the scalar-relativistic implementation of DIP-EOMCCSDT­(4*h*-2*p*).^20^ The DIP-EOMCCSD­(T)­(*ã*)­(4*h*-2*p*) approximation developed in this work, which can be performed using computational steps that scale as $N^{7}$ with the system size N , is capable of delivering DIPs for the relevant states of (Cl_2_)^2+^ in agreement with their counterparts obtained with the much more expensive DIP-EOMCCSDT­(4*h*-2*p*) and DIP-EOMCCSD­(T)­(a)­(4*h*-2*p*) approaches. Therefore, we rely on the DIP-EOMCCSD­(T)­(*ã*)­(4*h*-2*p*)/CBS level of theory in order to investigate the remaining atomic and molecular systems of interest in this study.

**Table 2:** Comparison of DIP Values (in eV) of Cl_2_ Molecule in Different 4c-FNS-DIP-EOMCC Methods with Pre-Existing Experimental and Theoretical Results^a^

	CCSD(3*h*-1*p*)		CCSD(3*h*-1*p*)^b^	CCSD(4*h*-2*p*)	CCSDT(4*h*-2*p*)	CCSD(T)(a)(4*h*-2*p*)	CCSD(T)(*ã*)(4*h*-2*p*)	
state	dyall.av3z	dyall.av4z	dyall.av3z	dyall.av4z	dyall.av4z	dyall.av4z	dyall.av4z	expt.^74^
*X* ^3^Σ^–^	31.41	31.60	31.40	30.90	31.21	31.19	31.20	31.13
*a* ^1^Δ	31.91	32.09	31.91	31.41	31.71	31.70	31.72	31.74
*b* ^1^Σ^+^	32.41	32.59	32.29	31.79	32.09	32.07	32.10	32.12
*c* ^1^Σ^–^	33.32	33.51	33.32	32.86	33.14	33.13	33.13	32.97

a We adopt a shorthand notation in which the methods 4c-DIP-EOMCCSD­(3*h*-1*p*), 4c-DIP-EOMCCSD­(4*h*-2*p*), 4c-DIP-EOMCCSD­(T)­(*ã*)­(4*h*-2*p*), 4c-DIP-EOMCCSD­(T)­(a)­(4*h*-2*p*), and 4c-DIP-EOMCCSDT­(4*h*-2*p*) are abbreviated as CCSD­(3*h*-1*p*), CCSD­(4*h*-2*p*), CCSD­(T)­(*ã*)­(4*h*-2*p*), CCSD­(T)­(a)­(4*h*-2*p*), and CCSDT­(4*h*-2*p*), respectively.

b Canonical DIP-EOMCCSD­(3*h*-1*p*) results taken from ref 27, calculated in dyall.av3z basis set.

In the 4c-FNS-DIP-EOMCCSD­(T)­(a)­(4*h*-2*p*) and 4c-FNS-DIP-EOMCCSD­(T)­(*ã*)­(4*h*-2*p*) methods, the $\hat{T}$ _3_ contribution is treated perturbatively. Consequently, the reliability of these approaches is expected to deteriorate in situations where the electronic structure develops strong multireference character, for example, upon significant bond stretching. This behavior is well-known for related perturbative approaches,^75^ such as CCSD­[T] and CCSD­(T), which may fail when bonds are substantially stretched and the single reference determinant no longer provides a qualitatively correct description of the underlying *N*-electron system. A detailed discussion on the topic is available in the Supporting Information.

### 4.4 Benchmarking: Atoms and Molecules

The benchmark systems considered here are closed-shell atoms and simple diatomic molecules that are well described within a single-reference framework and do not exhibit strong vibronic coupling. The present benchmarking primarily assesses the accuracy of the electronic-structure treatment. We note that for systems with strong electron–nuclear coupling, the DIP-EOMCC calculations performed in this work would not be sufficient. Furthermore, in systems characterized by stronger multireference correlations, the perturbative methods like DIP-EOMCCSD­(T)­(a)­(4*h*-2*p*) or DIP-EOMCCSD­(T)­(*ã*)­(4*h*-2*p*) may become unreliable, and thus turning to nonperturbative, multireference, or active-orbital-based treatments may be more appropriate.

In order to benchmark the performance of DIP-EOMCCSD­(T)­(*ã*)­(4*h*-2*p*) method, we have calculated the DIPs of the series of inert gas atoms Ar–Rn. The calculated DIP values along with their experimental counterparts are presented in Table 3. Key statistical parameters, including the maximum absolute deviation (MAD), mean absolute error (MAE), standard deviation (STD), and root-mean-square deviation (RMSD), are also included in Table 3. The experimental DIP value of Rn is less reliable, reported up to one significant digit, so it has been excluded from the statistical analysis. Using the dyall.av3z basis, the DIPs computed with DIP-EOMCCSD­(3*h*-1*p*) show good agreement with the available experimental results,^76^ with MAD and RMSD values of 0.26 and 0.14 eV, respectively. However, after performing CBS extrapolations, the errors in the DIPs obtained with DIP-EOMCCSD­(3*h*-1*p*) relative to experiment significantly increase, resulting in MAD and RMSD values of 0.47 and 0.35 eV, respectively. The increase in errors at the DIP-EOMCCSD­(3*h*-1*p*) level with the size of the basis is consistent with the trend observed for Cl_2_ discussed in the previous section. When the DIP-EOMCCSD­(3*h*-1*p*)/CBS method is replaced by its higher-level DIP-EOMCCSD­(T)­(*ã*)­(4*h*-2*p*)/CBS counterpart, the MAD and RMSD values relative to experiment for the DIPs reported in Table 3 reduce to 0.11 and 0.04 eV, respectively.

**Table 3:** Errors in DIP Energies (in eV) of Ar, Kr, Xe, and Rn Atoms with Respect to Experiment^a^

	state	CCSD(3*h*-1*p*)	CCSD(3*h*-1*p*)	CCSD(T)(*ã*)(4*h*-2*p*)	
atom		dyall.av3z	CBS	CBS	expt.^76^
Ar	^3^ *P*_2_	–0.01	0.40	–0.02	43.39
	^3^ *P*_1_	0.00	0.38	–0.01	43.53
	^3^ *P*_0_	0.01	0.40	–0.01	43.58
	^1^ *D*_2_	0.06	0.41	0.02	45.13
	^1^ *S*_0_	0.14	0.47	0.11	47.51
Kr	^3^ *P*_2_	–0.13	0.31	–0.02	38.36
	^3^ *P*_1_	–0.12	0.33	–0.02	38.92
	^3^ *P*_0_	–0.09	0.35	0.01	39.02
	^1^ *D*_2_	–0.05	0.32	0.01	40.18
	^1^ *S*_0_	–0.01	0.38	0.07	42.46
Xe	^3^ *P*_2_	–0.25	0.25	0.01	33.11
	^3^ *P*_1_	–0.26	0.26	–0.02	34.32
	^3^ *P*_0_	–0.20	0.29	0.01	34.11
	^1^ *D*_2_	–0.18	0.27	0.03	35.23
	^1^ *S*_0_	–0.13	0.31	0.06	37.58
Rn	^3^ *P*_2_	0.09	0.53	0.18	29.4
MAD^b^		0.26	0.47	0.11	
MAE^b^		0.11	0.34	0.03	
STD^b^		0.12	0.06	0.04	
RMSD^b^		0.14	0.35	0.04	

a We adopt a shorthand notation in which DIP-EOMCCSD­(3*h*-1*p*) and DIP-EOMCCSD­(T)­(*ã*)­(4*h*-2*p*) are abbreviated as CCSD­(3*h*-1*p*) and CCSD­(T)­(*ã*)­(4*h*-2*p*), respectively.

b Rn values were excluded from the statistical analysis.

To further demonstrate the capability of the present approach, we have benchmarked the 4c-FNS-DIP-EOMCCSD­(3*h*-1*p*) and FNS-DIP-EOMCCSD­(T)­(*ã*)­(4*h*-2*p*) methods for the closed-shell *s* ^2^ *d* ^10^ Group 12 atoms (Zn, Cd, and Hg). The computed results are compared with experimental values^76^ serving as reference (see Table S4). The 4c-FNS-DIP-EOMCCSD­(T)­(*ã*)­(4*h*-2*p*) results show a significant improvement over the 4c-FNS-DIP-EOMCCSD­(3*h*-1*p*) results for all states of Hg. In contrast, for Zn and Cd, the improvement is observed only for the first doubly ionized states. A more detailed analysis, including a possible reassessment of the experimental peak assignments in the double-ionization spectra of Zn and Cd, may therefore be required. Such an investigation is beyond the scope of the present study.

The DIP-EOMCCSD­(T)­(*ã*)­(4*h*-2*p*) approach is also applied to obtain the valence DIPs of Cl_2_, Br_2_, HBr, and HI, which have been considered in previous works.^20,27,33^ Similar to atoms, the DIP values at the DIP-EOMCCSD­(3*h*-1*p*)/dyall.av3z level show good agreement with experiment,^74,77–79^ with a MAD of 0.35 eV and RMSD of 0.19 eV obtained for the valence states of (Cl_2_)^2+^, (Br_2_)^2+^, (HBr)^2+^, and (HI)^2+^ considered in Table 4. Consistent with previous observations, the errors obtained with DIP-EOMCCSD­(3*h*-1*p*) increase when the basis set size is increased and extrapolated toward the CBS limit. Indeed, the DIPs computed with DIP-EOMCCSD­(3*h*-1*p*)/CBS are less accurate relative to experiment than their counterparts obtained using the dyall.av3z basis, with MAD and RMSD values of 0.66 and 0.44 eV, respectively. Larger errors are observed, especially for Cl_2_ and HBr. The errors relative to experiment significantly reduce when the DIP-EOMCCSD­(3*h*-1*p*)/CBS method is replaced by the higher-level DIP-EOMCCSD­(T)­(*ã*)­(4*h*-2*p*)/CBS approach. In particular, when using DIP-EOMCCSD­(T)­(*ã*)­(4*h*-2*p*)/CBS, the DIPs for Cl_2_, Br_2_, HBr, and HI are characterized by MAD and RMSD values relative to experiment of 0.30 and 0.15 eV, respectively. When examining the accuracy of the DIP-EOMCCSD­(T)­(*ã*)­(4*h*-2*p*)/CBS results reported in Table 4 relative to experiment, we observe slighly larger errors for molecules than for the atoms considered in Table 3. This difference in performance may be due to the effects of molecular vibrations, which are not included in the present DIP-EOMCC calculations.

**Table 4:** Errors in DIP Energies (in eV) of Cl_2_, Br_2_, HBr, and HI Molecules with Respect to Experiment^a^

	state	CCSD(3*h*-1*p*)	CCSD(3*h*-1*p*)	CCSD(T)(*ã*)(4*h*-2*p*)	
molecule		dyall.av3z	CBS	CBS	expt.^74,77–79^
Cl_2_	*X* ^3^Σ^–^	0.28	0.59	0.21	31.13
	*a* ^1^Δ	0.17	0.46	0.11	31.74
	*b* ^1^Σ^+^	0.29	0.58	0.13	32.12
	*c* ^1^Σ^–^	0.35	0.66	0.29	32.97
Br_2_	*A* 0_*g*_	–0.05	0.26	0.02	28.39
	*A* 1_*g*_	–0.07	0.26	–0.04	28.53
	*A* 2_*g*_	0.07	0.40	0.02	28.91
	*A* 0_*g*_	–0.03	0.28	–0.02	29.38
HBr	*X* ^3^Σ^–^	0.17	0.51	0.13	32.62
	*a* ^1^Δ	0.26	0.53	0.24	33.95
	*b* ^1^Σ^+^	0.30	0.59	0.30	35.19
HI	*X* ^3^Σ_0_^–^	–0.14	0.26	0.02	29.15
	*A* ^3^Σ_1_^–^	–0.14	0.28	–0.02	29.37
	*a* ^1^Δ	–0.07	0.29	0.07	30.39
	*b* ^1^Σ^+^	0.01	0.36	0.12	31.64
MAD		0.35	0.66	0.30	
MAE		0.16	0.42	0.12	
STD		0.17	0.15	0.11	
RMSD		0.19	0.44	0.15	

a We adopt a shorthand notation in which DIP-EOMCCSD­(3*h*-1*p*) and DIP-EOMCCSD­(T)­(*ã*)­(4*h*-2*p*) are abbreviated as CCSD­(3*h*-1*p*) and CCSD­(T)­(*ã*)­(4*h*-2*p*), respectively.

The Br_2_ molecule requires special attention as the previous application of the DIP-EOMCCSDT­(4*h*-2*p*) approach based on the one-electron SFX2C framework (SFX2C1e) resulted in non-negligible errors.^20^ Table 5 presents the DIP values corresponding to the first ten states of (Br_2_)^2+^, in which we use the Λ–*S* coupling notation for electronic states, similar to that used by Fleig et al.^77^ The previous study by Pal and co-workers^27^ only reported four of these states. Our previous SFX2C1e-based DIP-EOMCCSDT­(4*h*-2*p*) study^20^ has not been able to distinguish between the lowest *A* 0_*g*_ and *A* 1_*g*_ states due to the neglect of spin–orbit coupling in the SFX2C1e calculations. The DIP-EOMCCSD­(T)­(*ã*)­(4*h*-2*p*) method based on a 4c-DC Hamiltonian can accurately resolve the lowest *A* 0_*g*_ and *A* 1_*g*_ states in agreement with experiment. The next two states, *A* 2_*g*_ and *A* 0_*g*_, also show good agreement with the experiment. As shown by Fleig et al.,^77^ the experimentally observed broad band at 30.3 eV contains contributions from a group of electronic states with energies ranging between 30.1–30.5 eV, which complicates the comparison between individual states and experiment. However, for all the states considered in Table 5, the DIP-EOMCCSD­(T)­(*ã*)­(4*h*-2*p*) method gives better agreement with experiment than the previously reported DIP-EOMCCSD­(3*h*-1*p*) values of ref 27. It is also worth noting that the DIP-EOMCCSD­(T)­(*ã*)­(4*h*-2*p*)/CBS results reported in Table 5 are in excellent agreement with the relativistic multireference CI (MRCI) results^77^ reported by Fleig et al. for all ten electronic states of (Br_2_)^2+^.

**Table 5:** Comparison of DIP-EOMCCSD­(T)­(*ã*)­(4*h*-2*p*)/CBS DIP Values of Br_2_ with Experiment and Previous Theoretical Results

state	CCSD(3*h*-1*p*)^a^	CCSD(T)(*ã*)(4*h*-2*p*)	MRCI^b^	expt.^b^
*A* 0_*g*_		28.41	28.39	28.39
*A* 1_*g*_	28.47	28.49	28.54	28.53
*A* 2_*g*_	29.04	28.93	29.01	28.91
*A* 0_*g*_	29.52	29.36	29.45	29.38
*B* 0_*u*_		29.77	29.78	
*B* 3_*u*_		29.80	29.81	
*B* 2_*u*_	29.79	30.16	30.16	30.30
*B* 1_*u*_		30.24	30.24	
*B* 0_*u*_		30.47	30.50	
*B* 1_*u*_		30.51	30.52	

a Taken from ref 27.

b Taken from ref 77.

### 4.5 Effect of the Treatment of Relativity

It is important to investigate the impact of different levels of treating the relativistic Hamiltonian on the DIPs obtained in the DIP-EOMCC calculations. Table 6 presents a comparison of DIPs characterizing Ar, Kr, Xe and Rn atoms using NR, SFX2C1e, and 4c-DC Hamiltonians alongside the experimentally determined results. All DIPs are obtained with the DIP-EOMCCSD­(T)­(*ã*)­(4*h*-2*p*)/CBS calculations.

**Table 6:** Comparison of DIP Energies (in eV) of Ar, Kr, Xe, and Rn Atoms Using DIP-EOMCCSD­(T)­(*ã*)­(4*h*-2*p*) at CBS Level with NR and Various Relativistic Hamiltonians

atom	state	NR(CBS)	SFX2C1e(CBS)	4c-DC(CBS)	4c-DCG(CBS)	4c-DCB(CBS)	expt.^76^
Ar	^3^ *P*_2_	43.47	43.44	43.37	43.37	43.37	43.39
	^3^ *P*_1_	43.47	43.44	43.51	43.49	43.51	43.53
	^3^ *P*_0_	43.47	43.44	43.58	43.56	43.56	43.58
	^1^ *D*_2_	45.17	45.14	45.15	45.13	45.15	45.13
	^1^ *S*_0_	47.63	47.61	47.62	47.62	47.62	47.51
Kr	^3^ *P*_2_	38.65	38.64	38.34	38.33	38.33	38.36
	^3^ *P*_1_	38.65	38.64	38.90	38.88	38.88	38.92
	^3^ *P*_0_	38.65	38.64	39.03	39.00	39.02	39.02
	^1^ *D*_2_	40.11	40.11	40.19	40.16	40.15	40.18
	^1^ *S*_0_	42.34	42.38	42.53	42.51	42.51	42.46
Xe	^3^ *P*_2_	33.74	33.78	33.11	33.11	33.10	33.11
	^3^ *P*_1_	33.74	33.78	34.30	34.26	34.28	34.32
	^3^ *P*_0_	33.74	33.78	34.13	34.11	34.11	34.11
	^1^ *D*_2_	34.91	34.97	35.26	35.23	35.23	35.23
	^1^ *S*_0_	36.81	36.95	37.64	37.59	37.61	37.58
Rn	^3^ *P*_2_	31.57	31.78	29.58	29.56	29.57	29.4
MAD^a^		0.77	0.67	0.11	0.11	0.11	
MAE^a^		0.28	0.26	0.03	0.03	0.03	
STD^a^		0.35	0.32	0.04	0.04	0.04	
RMSD^a^		0.36	0.33	0.04	0.04	0.04	

a Rn values were excluded from the statistical analysis.

The NR calculations show large errors relative to experiment, with MAD and RMSD values of 0.77 and 0.36 eV, respectively. As one might expect, the largest error is observed for the heaviest atom, Rn, but it is excluded from the statistical analysis due to the unreliability of the experimental DIP energy. The use of scalar relativity, however, slightly improves the situation. As shown in Table 6, the errors relative to experiment characterizing the DIPs of Ar–Rn decrease when relativistic effects are treated using the SFX2C1e approach, with MAD and RMSD values of 0.67 and 0.33 eV, respectively. The use of 4c-DC Hamiltonian reduces the errors significantly and provides results with MAD and RMSD of 0.11 and 0.04, respectively. Based on the results for Ar–Rn reported in Table 6, one must use a more complete 4c-DC Hamiltonian in order to obtain DIP values that agree with experiment. The inclusion of the Gaunt and Breit corrections have negligible effect on the DIP values considered in Table 6.

When considering the Cl_2_, Br_2_, HBr, and HI molecules, we do not observe the same behavior in DIP values as we do for atoms when the treatment of relativity is improved. In particular, the MAD and RMSD values characterizing the DIPs computed with DIP-EOMCCSD­(T)­(*ã*)­(4*h*-2*p*)/CBS reported in Table 7, which are 0.30 and 0.15 eV, respectively, are similar to their counterparts obtained with the SFX2C1e treatments. As in the case for the Ar–Rn atoms, the inclusion of the Gaunt and Breit corrections is negligible.

**Table 7:** Comparison of DIP Energies (in eV) of Cl_2_, Br_2_, HBr, and HI Molecules Using DIP-EOMCCSD­(T)­(*ã*)­(4*h*-2*p*) at CBS Level with NR and Various Relativistic Hamiltonians

molecule	state	NR(CBS)	SFX2C1e(CBS)	4c(CBS)	DCG(CBS)	DCB(CBS)	expt.^74,77–79^
Cl_2_	*X* ^3^Σ^–^	31.39	31.31	31.34	31.32	31.32	31.13
	*a* ^1^Δ	31.87	31.83	31.85	31.84	31.83	31.74
	*b* ^1^Σ^+^	32.26	32.22	32.25	32.23	32.23	32.12
	*c* ^1^Σ^–^	33.32	33.28	33.26	33.24	33.24	32.97
Br_2_	*A* 0_*g*_	28.56	28.51	28.41	28.40	28.40	28.39
	*A* 1_*g*_	28.56	28.51	28.49	28.48	28.48	28.53
	*A* 2_*g*_	29.00	28.95	28.93	28.92	28.91	28.91
	*A* 0_*g*_	29.34	29.29	29.36	29.38	29.38	29.38
HBr	*X* ^3^Σ^–^	32.89	32.85	32.75	32.74	32.75	32.62
	*a* ^1^Δ	34.23	34.21	34.19	34.18	34.17	33.95
	*b* ^1^Σ^+^	35.49	35.48	35.49	35.48	35.49	35.19
HI	*X* ^3^Σ_0_^–^	29.40	29.39	29.17	29.16	29.18	29.15
	*A* ^3^Σ_1_^–^	29.40	29.39	29.35	29.33	29.34	29.37
	*a* ^1^Δ	30.50	30.50	30.46	30.45	30.44	30.39
	*b* ^1^Σ^+^	31.58	31.58	31.76	31.74	31.75	31.64
MAD		0.34	0.30	0.30	0.29	0.30	
MAE		0.17	0.14	0.12	0.11	0.11	
STD		0.16	0.12	0.11	0.11	0.11	
RMSD		0.20	0.17	0.15	0.14	0.14	

## 5 Conclusions

In this work, we have developed and tested a suite of 4c relativistic DIP-EOMCC methods incorporating up to 4*h*-2*p* excitations and three-body clusters. We have extended the original DIP-EOMCCSDT­(4*h*-2*p*) method and its perturbative DIP-EOMCCSD­(T)­(a)­(4*h*-2*p*) approximation to the fully relativistic regime. In addition, we have introduced a new, low-cost approximation to DIP-EOMCCSD­(T)­(a)­(4*h*-2*p*), abbreviated as DIP-EOMCCSD­(T)­(*ã*)­(4*h*-2*p*), which is capable of accurately reproducing the DIPs obtained with DIP-EOMCCSD­(T)­(a)­(4*h*-2*p*) and DIP-EOMCCSDT­(4*h*-2*p*) using much less expensive computational steps that scale as $N^{7}$ . This makes the DIP-EOMCCSD­(T)­(*ã*)­(4*h*-2*p*) approach a practical tool for studying the DIPs of atomic and molecular systems using realistic basis sets and relativistic Hamiltonians. We have also combined all of these approaches with the FNS truncation scheme and showed that this allows us to significantly reduce the computational cost without compromising accuracy. Benchmark calculations on inert gas atoms (Ar–Rn) and diatomics (Cl_2_, Br_2_, HBr, HI) demonstrate that the proposed DIP-EOMCCSD­(T)­(*ã*)­(4*h*-2*p*) method produces DIPs in good agreement with experimental results after CBS extrapolations are performed. It was also found that the calculated DIP values are very sensitive to the basis set size, and in order to compare with experiment, large basis sets or CBS extrapolations are necessary. The need for large basis sets highlights the usefulness of the FNS scheme, as we are able to handle larger calculations in QZ-level basis sets without running into prohibitive computational or memory bottlenecks. Furthermore, we have benchmarked the effects of NR and scalar relativistic treatments against their complete 4c-DC parent, and showed that only the 4c-DC Hamiltonian is capable of providing robust and accurate DIPs of atomic and molecular systems containing heavier elements.

## Supplementary Material



## Data Availability

The data that support the findings of this study are available within the article.

## Notes

DIP values in DZ, TZ, and QZ basis used for CBS extrapolation and programmable expressions for DIP-EOMCCSD­(T)­(*ã*)­(4*h*-2*p*) (PDF)
