# Supplementary material for: Reduced-Cost Four-Component Relativistic Double Ionization Potential Equation-of-Motion Coupled-Cluster Approaches with 4‑Hole–2-Particle Excitations and Three-Body Clusters
Source: J Chem Theory Comput. 2026 Mar 27;22(7):3233–46. doi: 10.1021/acs.jctc.5c01791 (PMC13085238; doi:10.1021/acs.jctc.5c01791)
Supplement: Supplementary file 1 [file ct5c01791_si_001.pdf]

Supporting Information: Reduced-Cost Four-Component Relativistic Double Ionization Potential Equation-of-Motion Coupled-Cluster Approaches with 4-Hole–2-Particle Excitations and Three-Body Clusters

Tamoghna Mukhopadhyay,[†] Madhubani Mukherjee,[†] Karthik Gururangan,[‡] Piotr Piecuch,[*,‡,¶] and Achintya Kumar Dutta[*,†,§]

†*Department of Chemistry, Indian Institute of Technology Bombay, Powai, Mumbai 400076, India*

‡*Department of Chemistry, Michigan State University, East Lansing, MI 48824, USA*

¶*Department of Physics and Astronomy, Michigan State University, East Lansing, MI 48824, USA*

§*Department of Inorganic Chemistry, Faculty of Natural Sciences, Comenius University Bratislava Ilkovičova 6, Mlynská dolina 842 15 Bratislava, Slovakia*

E-mail: piecuch@chemistry.msu.edu; achintya@chem.iitb.ac.in

List of Tables

S1 The expressions of $\bar{H}$ intermediates for DIP-EOMCCSD(T)($\tilde{a}$)(4h-2p) approach . . . 4

S2 The intermediates defined to account for contributions in DIP-EOMCCSD(T)($\tilde{a}$)(4h-2p) sigma equations due to three- and four-body components of $\bar{H}$. 4

S3 4c-FNS-DIP-EOM-CCSD (3h-1p) energies (in eV) of Cl_2, Br_2. HBr and HI molecules at CBS limit extrapolated using different CBS extrapolation methods at FNS threshold of $10^{-4.5}$. In the following table, we adopt at shorthand notation in which Feller, Helgaker, Lesiuk, Martin, Peterson and Dunning are abbreviated as F, H, L, M, P and D, respectively. . . . 5

S4 The comparison of errors in DIP energies (in eV) of Zn, Cd and Hg atoms (in dyall.av4z basis set) with respect to the experiment along with experimental DIP values (in eV). In the following table, we adopt a shorthand notation in which 4c-FNS-DIP-EOMCCSD(T)($\tilde{a}$)(4h-2p) is abbreviated as CCSD(T)($\tilde{a}$)(4h-2p), respectively. . . . 6

S5 DIP energies (in eV) of Ar, Kr, Xe and Rn atoms using different levels of theories at FNS threshold of $10^{-4.5}$ in dyall.av2z basis set. In the following table, we adopt at shorthand notation in which DIP-EOMCCSD(3h-1p) and DIP-EOMCCSD(T)($\tilde{a}$)(4h-2p) are abbreviated as CCSD(3h-1p) and CCSD(T)($\tilde{a}$)(4h-2p), respectively. . . . 7

S6 DIP energies (in eV) of Cl_2, Br_2. HBr and HI molecules using different levels of theories at FNS threshold of $10^{-4.5}$ in dyall.av2z basis set. In the following table, we adopt at shorthand notation in which DIP-EOMCCSD(3h-1p) and DIP-EOMCCSD(T)($\tilde{a}$)(4h-2p) are abbreviated as CCSD(3h-1p) and CCSD(T)($\tilde{a}$)(4h-2p), respectively. . . . 8

S7 DIP energies (in eV) of Ar, Kr, Xe and Rn atoms using different levels of theories at FNS threshold of $10^{-4.5}$ in dyall.av3z basis set. In the following table, we adopt at shorthand notation in which DIP-EOMCCSD(3h-1p) and DIP-EOMCCSD(T)($\tilde{a}$)(4h-2p) are abbreviated as CCSD(3h-1p) and CCSD(T)($\tilde{a}$)(4h-2p), respectively. 9

S8 DIP energies (in eV) of Cl_2, Br_2. HBr and HI molecules using different levels of theories at FNS threshold of $10^{-4.5}$ in dyall.av3z basis set. In the following table, we adopt at shorthand notation in which DIP-EOMCCSD(3h-1p) and DIP-EOMCCSD(T)($\tilde{a}$)(4h-2p) are abbreviated as CCSD(3h-1p) and CCSD(T)($\tilde{a}$)(4h-2p), respectively. 10

S9 DIP energies (in eV) of Ar, Kr, Xe and Rn atoms using different levels of theories at FNS threshold of $10^{-4.5}$ in dyall.av4z basis set. In the following table, we adopt at shorthand notation in which DIP-EOMCCSD(3h-1p) and DIP-EOMCCSD(T)($\tilde{a}$)(4h-2p) are abbreviated as CCSD(3h-1p) and CCSD(T)($\tilde{a}$)(4h-2p), respectively. 11

S10 DIP energies (in eV) of Cl_2, Br_2. HBr and HI molecules using different levels of theories at FNS threshold of $10^{-4.5}$ in dyall.av4z basis set. In the following table, we adopt at shorthand notation in which DIP-EOMCCSD(3h-1p) and DIP-EOMCCSD(T)($\tilde{a}$)(4h-2p) are abbreviated as CCSD(3h-1p) and CCSD(T)($\tilde{a}$)(4h-2p), respectively. 12

Table S1: The expressions of $\bar{H}$ intermediates for DIP-EOMCCSD(T)($\tilde{a}$)(4h-2p) approach

Component of $\overline{H}_N$	Expression
h^{e}_{m}	$f^{e}_{m} + v^{ef}_{mn} t^{n}_{f}$
$\bar{h}^{i}_{j}$	$f^{i}_{j} + \bar{h}^{e}_{j} t^{i}_{e} + v^{ie}_{jm} t^{m}_{e} + \frac{1}{2} v^{ef}_{jn} t^{in}_{ef}$
$\bar{h}^{b}_{a}$	$f^{b}_{a} - \bar{h}^{b}_{m} t^{m}_{a} + v^{be}_{am} t^{m}_{e} - \frac{1}{2} v^{bf}_{mn} t^{mn}_{af}$
$\bar{h}^{ef}_{mn}$	v^{ef}_{mn}
$\bar{h}^{ef}_{am}$	$v^{ef}_{am} - v^{fe}_{mn} t^{n}_{a}$
$\bar{h}^{ie}_{mn}$	$v^{ie}_{mn} + v^{fe}_{mn} t^{i}_{f}$
$\bar{h}^{ef}_{ab}$	$v^{ef}_{ab} + \frac{1}{2} v^{ef}_{mn} \tau^{mn}_{ab} - \mathscr{A}_{ab} v^{ef}_{am} t^{m}_{b}$
$\bar{h}^{ij}_{mn}$	$v^{ij}_{mn} + \frac{1}{2} v^{ef}_{mn} \tau^{ij}_{ef} + \mathscr{A}^{ij} v^{je}_{nm} t^{i}_{e}$
$\bar{h}^{ie}_{am}$	$v^{ie}_{am} + v^{fe}_{am} t^{i}_{f} - \bar{h}^{ie}_{nm} t^{n}_{a} + v^{ef}_{mn} t^{in}_{af}$
$\bar{h}^{ij}_{am}$	$v^{ij}_{am} + \bar{h}^{e}_{m} t^{ij}_{ae} - \bar{h}^{ij}_{nm} t^{n}_{a} + \frac{1}{2} v^{ef}_{am} t^{ij}_{ef} + \mathscr{A}^{ij} (\bar{h}^{jf}_{mn} t^{in}_{af} + \chi'^{ie}_{am} t^{j}_{e}) + v^{ef}_{mn} t^{ijn}_{aef}$
$\bar{h}^{ie}_{ab}$	$v^{ie}_{ab} - \bar{h}^{e}_{m} t^{im}_{ab} + v^{ef}_{ab} t^{i}_{f} + \frac{1}{2} \bar{h}^{ie}_{mn} t^{mn}_{ab} - \mathscr{A}_{ab} (\chi^{ie}_{am} t^{m}_{b} - v^{ef}_{bn} t^{in}_{af}) - v^{ef}_{mn} t^{imn}_{abf}$
χ'^{ie}_{am}	$v^{ie}_{am} + \frac{1}{2} v^{ef}_{am} t^{i}_{e}$
χ^{ie}_{am}	$\bar{h}^{ie}_{am} + \frac{1}{2} \bar{h}^{ie}_{nm} t^{n}_{a}$
τ^{ij}_{ab}	$t^{ij}_{ab} + \mathscr{A}^{ij} t^{i}_{a} t^{j}_{b}$

Table S2: The intermediates defined to account for contributions in DIP-EOMCCSD(T)($\tilde{a}$)(4h-2p) sigma equations due to three- and four-body components of $\bar{H}$.

Intermediate	Expression
$I'^{ie}(\mu)$	$\frac{1}{2} \bar{h}^{ie}_{mn} r^{mn}(\mu) - \frac{1}{2} \bar{h}^{fe}_{mn} r^{inm}{}_{f}(\mu)$
$I^{ijk}{}_{m}(\mu)$	$\mathscr{A}^{ijk} [\frac{1}{2} \bar{h}^{ke}_{nm} r^{ijm}{}_{e}(\mu) - \frac{1}{2} \bar{h}^{ik}_{nm} r^{nj}(\mu) + \frac{1}{12} \bar{h}^{ef}_{mn} r^{ijkn}{}_{ef}(\mu)]$
$I^{ije}{}_{c}(\mu)$	$\bar{h}^{fe}_{cm} r^{ijm}{}_{e}(\mu) + \frac{1}{2} I^{fe}(\mu) t^{ij}_{cf} + \mathscr{A}^{ij} [\bar{h}^{ie}_{cm} r^{mj}(\mu) + \frac{1}{2} \bar{h}^{ie}_{nm} r^{njm}{}_{c}(\mu)] - \frac{1}{2} \bar{h}^{ef}_{mn} r^{ijmn}{}_{cf}(\mu)$

Table S3: 4c-FNS-DIP-EOM-CCSD ($3h$-$1p$) energies (in eV) of Cl_2, Br_2. HBr and HI molecules at CBS limit extrapolated using different CBS extrapolation methods at FNS threshold of $10^{-4.5}$. In the following table, we adopt at shorthand notation in which Feller, Helgaker, Lesiuk, Martin, Peterson and Dunning are abbreviated as F, H, L, M, P and D, respectively.

Molecule	States	DIP			
		F-H	F-L	M	P-D
Cl_2	$X\ ^3\Sigma^-$	31.78	31.82	31.75	31.72
	$a\ ^1\Delta$	32.26	32.30	32.23	32.20
	$b\ ^1\Sigma^+$	32.75	32.77	32.72	32.70
	$c\ ^1\Sigma^-$	33.69	33.74	33.66	33.63
Br_2	$A\ 0_g$	28.72	28.77	28.68	28.65
	$A\ 1_g$	28.86	28.92	28.82	28.79
	$A\ 2_g$	29.38	29.44	29.34	29.31
	$A\ 0_g$	29.73	29.78	29.69	29.66
	$B\ 0_u$	30.08	30.14	30.04	30.01
	$B\ 3_u$	30.11	30.17	30.08	30.04
	$B\ 2_u$	30.45	30.51	30.42	30.38
	$B\ 1_u$	30.53	30.60	30.50	30.46
	$B\ 0_u$	30.79	30.85	30.76	30.72
	$B\ 1_u$	30.82	30.88	30.79	30.75
HBr	$X\ ^3\Sigma^-$	33.19	33.24	33.16	33.13
	$a\ ^1\Delta$	34.54	34.59	34.51	34.48
	$b\ ^1\Sigma^+$	35.84	35.88	35.81	35.78
HI	$X\ ^3\Sigma_0^-$	29.49	29.55	29.45	29.41
	$A\ ^3\Sigma_1^-$	29.73	29.79	29.69	29.65
	$a\ ^1\Delta$	30.75	30.82	30.72	30.68
	$b\ ^1\Sigma^+$	32.07	32.12	32.04	32.00

Table S4: The comparison of errors in DIP energies (in eV) of Zn, Cd and Hg atoms (in dyall.av4z basis set) with respect to the experiment along with experimental DIP values (in eV). In the following table, we adopt a shorthand notation in which 4c-FNS-DIP-EOMCCSD(T)($\tilde{a}$)(4h-2p) is abbreviated as CCSD(T)($\tilde{a}$)(4h-2p), respectively.

Atom	States	CCSD(3h-1p)	CCSD(T)($\tilde{a}$)(4h-2p)	Expt.
Zn	$^1S_0$	-0.378	-0.242	27.359
	$^3D_3$	-0.078	0.279	37.042
	$^3D_2$	-0.071	0.293	37.188
	$^3D_1$	-0.054	0.306	37.383
	$^1D_2$	-0.088	0.251	37.712
Cd	$^1S_0$	-0.238	0.007	25.902
	$^3D_3$	0.041	0.459	35.877
	$^3D_2$	0.041	0.463	36.113
	$^3D_1$	0.074	0.492	36.592
	$^1D_2$	0.051	0.459	36.921
Hg	$^1S_0$	-0.312	-0.214	29.194
	$^3D_3$	0.300	-0.013	34.507
	$^3D_2$	0.280	-0.005	34.901
	$^3D_1$	0.302	-0.060	36.435
	$^1D_2$	0.288	-0.055	36.768
MAD		0.378	0.492	
MAE		0.173	0.342	
STD		0.219	0.223	
RMSD		0.212	0.377	

Table S5: DIP energies (in eV) of Ar, Kr, Xe and Rn atoms using different levels of theories at FNS threshold of $10^{-4.5}$ in dyall.av2z basis set. In the following table, we adopt at shorthand notation in which DIP-EOMCCSD($3h$-$1p$) and DIP-EOMCCSD(T)($\tilde{a}$)($4h$-$2p$) are abbreviated as CCSD($3h$-$1p$) and CCSD(T)($\tilde{a}$)($4h$-$2p$), respectively.

Atom	States	NR	SFX2C1e	CCSD($3h$-$1p$)	CCSD(T)($\tilde{a}$)($4h$-$2p$)		
					DC	DCG	DCB
Ar	$^3P_2$	42.69	42.66	43.00	42.59	42.58	42.58
	$^3P_1$	42.69	42.66	43.14	42.73	42.71	42.72
	$^3P_0$	42.69	42.66	43.20	42.79	42.77	42.77
	$^1D_2$	44.60	44.57	44.96	44.58	44.57	44.57
	$^1S_0$	46.91	46.90	47.19	46.91	46.90	46.90
Kr	$^3P_2$	37.90	37.89	37.90	37.60	37.59	37.59
	$^3P_1$	37.90	37.89	38.47	38.14	38.12	38.12
	$^3P_0$	37.90	37.89	38.58	38.24	38.22	38.23
	$^1D_2$	39.56	39.56	39.93	39.64	39.61	39.62
	$^1S_0$	41.65	41.70	42.07	41.85	41.84	41.84
Xe	$^3P_2$	32.92	32.97	32.50	32.33	32.32	32.32
	$^3P_1$	32.92	32.97	33.69	33.49	33.46	33.47
	$^3P_0$	32.92	32.97	33.51	33.33	33.31	33.32
	$^1D_2$	34.32	34.39	34.83	34.65	34.62	34.63
	$^1S_0$	36.03	36.21	37.00	36.90	36.86	36.87
Rn	$^3P_2$	30.74	31.01	29.18	28.94	28.93	28.93

Table S6: DIP energies (in eV) of Cl_2, Br_2. HBr and HI molecules using different levels of theories at FNS threshold of $10^{-4.5}$ in dyall.av2z basis set. In the following table, we adopt at shorthand notation in which DIP-EOMCCSD(3h-1p) and DIP-EOMCCSD(T)($\tilde{a}$)(4h-2p) are abbreviated as CCSD(3h-1p) and CCSD(T)($\tilde{a}$)(4h-2p), respectively.

Molecule	States	NR	SFX2C1e	CCSD(3h-1p)	CCSD(T)($\tilde{a}$)(4h-2p)		
					DC	DCG	DCB
Cl_2	$X\ ^3\Sigma^-$	30.69	30.66	31.05	30.69	30.70	30.70
	$a\ ^1\Delta$	31.27	31.24	31.58	31.24	31.23	31.23
	$b\ ^1\Sigma^+$	31.64	31.61	31.94	31.62	31.61	31.61
	$c\ ^1\Sigma^-$	32.70	32.66	32.99	32.65	32.65	32.65
Br_2	$A\ 0_g$	27.97	27.92	28.08	27.83	27.82	27.82
	$A\ 1_g$	27.97	27.92	28.19	27.90	27.89	27.89
	$A\ 2_g$	28.46	28.41	28.72	28.39	28.38	28.38
	$A\ 0_g$	28.79	28.73	29.09	28.86	28.84	28.84
	$B\ 0_u$	29.53	29.44	29.44	29.21	29.21	29.21
	$B\ 3_u$	29.69	29.61	29.48	29.26	29.26	29.26
	$B\ 2_u$	29.69	29.61	29.84	29.61	29.60	29.60
	$B\ 1_u$	29.69	29.61	29.91	29.69	29.67	29.68
	$B\ 0_u$	29.77	29.69	30.17	29.93	29.91	29.92
	$B\ 1_u$	29.77	29.69	30.19	29.96	29.94	29.94
HBr	$X\ ^3\Sigma^-$	32.13	32.09	32.34	32.06	32.05	32.05
	$a\ ^1\Delta$	33.70	33.67	33.92	33.67	33.65	33.65
	$b\ ^1\Sigma^+$	35.02	34.90	35.13	34.93	35.03	35.03
HI	$X\ ^3\Sigma_0^-$	28.52	28.52	28.52	28.32	28.30	28.31
	$A\ ^3\Sigma_1^-$	28.52	28.52	28.72	28.52	28.50	28.51
	$a\ ^1\Delta$	29.88	29.89	30.00	29.85	29.83	29.84
	$b\ ^1\Sigma^+$	30.90	30.97	31.21	31.10	31.07	31.08

Table S7: DIP energies (in eV) of Ar, Kr, Xe and Rn atoms using different levels of theories at FNS threshold of $10^{-4.5}$ in dyall.av3z basis set. In the following table, we adopt at shorthand notation in which DIP-EOMCCSD($3h$-$1p$) and DIP-EOMCCSD(T)($\tilde{a}$)($4h$-$2p$) are abbreviated as CCSD($3h$-$1p$) and CCSD(T)($\tilde{a}$)($4h$-$2p$), respectively.

Atom	States	NR	SFX2C1e	CCSD($3h$-$1p$)	CCSD(T)($\tilde{a}$)($4h$-$2p$)		
					DC	DCG	DCB
Ar	$^3P_2$	43.04	43.01	43.38	42.94	42.93	42.94
	$^3P_1$	43.04	43.01	43.53	43.08	43.07	43.07
	$^3P_0$	43.04	43.01	43.59	43.14	43.12	43.13
	$^1D_2$	44.80	44.77	45.19	44.78	44.77	44.77
	$^1S_0$	47.28	47.27	47.65	47.27	47.26	47.26
Kr	$^3P_2$	38.17	38.17	38.23	37.87	37.86	37.86
	$^3P_1$	38.17	38.17	38.80	38.42	38.40	38.40
	$^3P_0$	38.17	38.17	38.93	38.53	38.51	38.51
	$^1D_2$	39.69	39.70	40.12	39.77	39.75	39.76
	$^1S_0$	41.96	42.00	42.45	42.15	42.13	42.13
Xe	$^3P_2$	33.19	33.25	32.85	32.60	32.58	32.59
	$^3P_1$	33.19	33.25	34.06	33.77	33.74	33.74
	$^3P_0$	33.19	33.25	33.91	33.62	33.60	33.60
	$^1D_2$	34.43	34.51	35.05	34.78	34.75	34.76
	$^1S_0$	36.36	36.53	37.45	37.19	37.15	37.15
Rn	$^3P_2$	31.01	31.26	29.49	29.22	29.20	29.21

Table S8: DIP energies (in eV) of Cl_2, Br_2. HBr and HI molecules using different levels of theories at FNS threshold of $10^{-4.5}$ in dyall.av3z basis set. In the following table, we adopt at shorthand notation in which DIP-EOMCCSD($3h$-$1p$) and DIP-EOMCCSD(T)($\tilde{a}$)($4h$-$2p$) are abbreviated as CCSD($3h$-$1p$) and CCSD(T)($\tilde{a}$)($4h$-$2p$), respectively.

Molecule	States	NR	SFX2C1e	CCSD($3h$-$1p$)	CCSD(T)($\tilde{a}$)($4h$-$2p$)		
					DC	DCG	DCB
Cl_2	$X\ ^3\Sigma^-$	31.00	30.97	31.41	30.99	30.98	30.98
	$a\ ^1\Delta$	31.54	31.50	31.91	31.51	31.50	31.51
	$b\ ^1\Sigma^+$	31.93	31.90	32.41	31.85	31.87	31.87
	$c\ ^1\Sigma^-$	32.98	32.94	33.32	32.92	32.92	32.92
Br_2	$A\ 0_g$	28.22	28.17	28.34	28.08	28.07	28.07
	$A\ 1_g$	28.22	28.17	28.46	28.16	28.14	28.15
	$A\ 2_g$	28.67	28.62	28.98	28.61	28.59	28.60
	$A\ 0_g$	29.02	28.97	29.35	29.06	29.02	29.02
	$B\ 0_u$	29.75	29.68	29.68	29.43	29.43	29.43
	$B\ 3_u$	29.89	29.82	29.71	29.47	29.47	29.47
	$B\ 2_u$	29.89	29.82	30.07	29.82	29.81	29.81
	$B\ 1_u$	29.89	29.82	30.13	29.89	29.88	29.88
	$B\ 0_u$	29.96	29.88	30.41	30.14	30.12	30.13
	$B\ 1_u$	29.96	29.88	30.42	30.17	30.15	30.16
HBr	$X\ ^3\Sigma^-$	32.55	32.52	32.79	32.47	32.45	32.46
	$a\ ^1\Delta$	33.95	33.92	34.21	33.91	33.89	33.90
	$b\ ^1\Sigma^+$	35.21	35.20	35.49	35.22	35.20	35.21
HI	$X\ ^3\Sigma_0^-$	29.01	29.00	29.01	28.80	28.79	28.79
	$A\ ^3\Sigma_1^-$	29.01	29.00	29.23	28.96	28.94	28.94
	$a\ ^1\Delta$	30.17	30.18	30.32	30.14	30.12	30.13
	$b\ ^1\Sigma^+$	31.27	31.32	31.65	31.45	31.43	31.43

Table S9: DIP energies (in eV) of Ar, Kr, Xe and Rn atoms using different levels of theories at FNS threshold of $10^{-4.5}$ in dyall.av4z basis set. In the following table, we adopt at shorthand notation in which DIP-EOMCCSD($3h$-$1p$) and DIP-EOMCCSD(T)($\tilde{a}$)($4h$-$2p$) are abbreviated as CCSD($3h$-$1p$) and CCSD(T)($\tilde{a}$)($4h$-$2p$), respectively.

Atom	States	NR	SFX2C1e	CCSD($3h$-$1p$)	CCSD(T)($\tilde{a}$)($4h$-$2p$)		
					DC	DCG	DCB
Ar	$^3P_2$	43.31	43.28	43.63	43.21	43.20	43.20
	$^3P_1$	43.31	43.28	43.77	43.35	43.33	43.34
	$^3P_0$	43.31	43.28	43.83	43.41	43.39	43.39
	$^1D_2$	45.03	45.00	45.40	45.00	44.99	45.00
	$^1S_0$	47.50	47.48	47.86	47.49	47.48	47.48
Kr	$^3P_2$	38.47	38.46	38.50	38.16	38.15	38.15
	$^3P_1$	38.47	38.46	39.08	38.71	38.69	38.69
	$^3P_0$	38.47	38.46	39.20	38.83	38.81	38.82
	$^1D_2$	39.94	39.95	40.35	40.02	40.00	40.00
	$^1S_0$	42.19	42.23	42.69	42.39	42.36	42.36
Xe	$^3P_2$	33.53	33.57	33.16	32.91	32.90	32.90
	$^3P_1$	33.53	33.57	34.38	34.09	34.06	34.07
	$^3P_0$	33.53	33.57	34.21	33.93	33.91	33.91
	$^1D_2$	34.72	34.79	35.32	35.07	35.04	35.04
	$^1S_0$	36.63	36.79	37.72	37.46	37.42	37.43
Rn	$^3P_2$	31.35	31.57	29.76	29.44	29.42	29.43

Table S10: DIP energies (in eV) of Cl_2, Br_2. HBr and HI molecules using different levels of theories at FNS threshold of $10^{-4.5}$ in dyall.av4z basis set. In the following table, we adopt at shorthand notation in which DIP-EOMCCSD($3h$-$1p$) and DIP-EOMCCSD(T)($\tilde{a}$)($4h$-$2p$) are abbreviated as CCSD($3h$-$1p$) and CCSD(T)($\tilde{a}$)($4h$-$2p$), respectively.

Molecule	States	NR	SFX2C1e	CCSD($3h$-$1p$)	CCSD(T)($\tilde{a}$)($4h$-$2p$)		
					DC	DCG	DCB
Cl_2	$X\ ^3\Sigma^-$	31.24	31.18	31.60	31.20	31.19	31.19
	$a\ ^1\Delta$	31.74	31.70	32.09	31.72	31.71	31.71
	$b\ ^1\Sigma^+$	32.13	32.10	32.59	32.10	32.09	32.09
	$c\ ^1\Sigma^-$	33.19	33.15	33.51	33.13	33.12	33.12
Br_2	$A\ 0_g$	28.43	28.38	28.53	28.28	28.27	28.27
	$A\ 1_g$	28.43	28.38	28.66	28.36	28.35	28.35
	$A\ 2_g$	28.87	28.82	29.18	28.80	28.79	28.79
	$A\ 0_g$	29.22	29.16	29.54	29.24	29.24	29.24
	$B\ 0_u$	29.96	29.88	29.88	29.64	29.63	29.63
	$B\ 3_u$	30.10	30.02	29.91	29.67	29.67	29.67
	$B\ 2_u$	30.10	30.02	30.26	30.03	30.02	30.02
	$B\ 1_u$	30.10	30.02	30.33	30.10	30.08	30.09
	$B\ 0_u$	30.17	30.09	30.60	30.34	30.32	30.33
	$B\ 1_u$	30.17	30.09	30.62	30.38	30.36	30.36
HBr	$X\ ^3\Sigma^-$	32.76	32.73	33.00	32.64	32.63	32.64
	$a\ ^1\Delta$	34.12	34.10	34.38	34.08	34.07	34.07
	$b\ ^1\Sigma^+$	35.38	35.37	35.67	35.39	35.37	35.38
HI	$X\ ^3\Sigma^-_0$	29.26	29.24	29.26	29.03	29.02	29.03
	$A\ ^3\Sigma^-_1$	29.26	29.24	29.49	29.20	29.18	29.19
	$a\ ^1\Delta$	30.37	30.38	30.54	30.34	30.32	30.32
	$b\ ^1\Sigma^+$	31.46	31.52	31.87	31.64	31.62	31.63

S.1 Limitations of perturbative triples-based DIP-EOMCC methods

To analyze the performance of 4c-FNS-DIP-EOMCCSD(T)($\tilde{a}$)(4h-2p) in multi-reference situations, we studied the potential energy curves (PECs) in the bond dissociation region. The ground-state PEC of F_2 was computed using CCSD, CCSD(T)(a), and CCSDT methods (Figure S1a), while the $^3\Sigma_g^-$ doubly ionized state (F_2^{2+}) was studied using 4c-FNS-DIP-EOMCCSD(3h-1p), 4c-FNS-DIP-EOMCCSD(T)(a)(4h-2p), 4c-FNS-DIP-EOMCCSD(T)($\tilde{a}$)(4h-2p), and 4c-FNS-DIP-EOMCCSDT(4h-2p) methods with the dyall.v2z basis set (Figure S1b). For the neutral F_2 ground state, CCSD(T)(a) exhibits a clear breakdown at a bond length beyond 2.2 Å, where the perturbative triples correction becomes unreliable, whereas CCSDT shows the correct asymptotic trend. This behavior reflects the growing static correlation effects in the dissociation limit, where perturbative treatments of triples are no longer adequate.

A similar trend is observed for the doubly ionized state. Although all DIP-EOMCC energies decrease upon bond stretching, deviations between perturbative and full triples treatments become evident at large internuclear separations. The 4c-FNS-DIP-EOMCCSD(T)(a)(4h-2p) and 4c-FNS-DIP-EOMCCSD(T)($\tilde{a}$)(4h-2p) increasingly deviate from 4c-FNS-DIP-EOMCCSDT(4h-2p) in the stretched-bond region. The deviation is larger for the 4c-FNS-DIP-EOMCCSD(T)($\tilde{a}$)(4h-2p) variant, consistent with the additional perturbative approximation introduced in the DIP matrix–vector equations.

Importantly, near equilibrium geometries, where the electronic structure remains predominantly single-reference in character, both perturbative variants show excellent agreement with the full 4c-FNS-DIP-EOMCCSDT results.

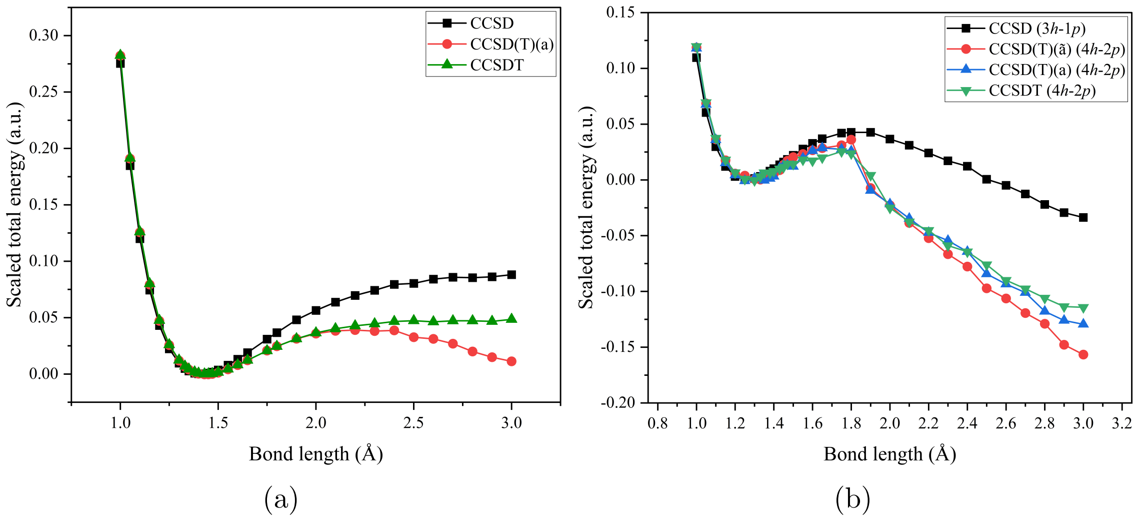

Figure S1: The potential energy curve of (a) ground state F_2 and (b) doubly ionized state F_2^{2+} using different 4c-FNS-DIP-EOMCC methods in dyall.v2z basis set.
